# Knock-out of 5-lipoxygenase in overexpressing tumor cells—consequences on gene expression and cellular function

**DOI:** 10.1038/s41417-022-00531-9

**Published:** 2022-09-16

**Authors:** Hannah Weisser, Tamara Göbel, G. Melissa Krishnathas, Marius Kreiß, Carlo Angioni, Duran Sürün, Dominique Thomas, Tobias Schmid, Ann-Kathrin Häfner, Astrid S. Kahnt

**Affiliations:** 1 Institute of Pharmaceutical Chemistry, Goethe University, Max-von-Laue-Straße 9, 60438 Frankfurt/Main, Germany; 2 Institute of Clinical Pharmacology, Pharmazentrum Frankfurt, ZAFES, Goethe University, Theodor-Stern-Kai 7, 60590 Frankfurt/Main, Germany; 3 Medical Systems Biology, Medical Faculty and University Hospital Carl Gustav Carus, TU Dresden, 01307 Dresden, Germany; 4 Fraunhofer Institute of Translational Medicine and Pharmacology ITMP, Theodor-Stern-Kai 7, 60596 Frankfurt/Main, Germany; 5 Institute of Biochemistry I, Faculty of Medicine, Goethe University, Theodor-Stern-Kai 7, 60590 Frankfurt, Germany

**Keywords:** Cell biology, Oncogenes, Gene expression

## Abstract

5-Lipoxygenase (5-LO), the central enzyme in the biosynthesis of leukotrienes, is frequently expressed in human solid malignancies even though the enzyme is not present in the corresponding healthy tissues. There is little knowledge on the consequences of this expression for the tumor cells regarding gene expression and cellular function. We established a knockout (KO) of 5-LO in different cancer cell lines (HCT-116, HT-29, U-2 OS) and studied the consequences on global gene expression using next generation sequencing. Furthermore, cell viability, proliferation, migration and multicellular tumor spheroid (MCTS) formation were studied in these cells. Our results show that 5-LO influences the gene expression and cancer cell function in a cell type-dependent manner. The enzyme affected genes involved in cell adhesion, extracellular matrix formation, G protein signaling and cytoskeleton organization. Furthermore, absence of 5-LO elevated TGFβ_2_ expression in HCT-116 cells while MCP-1, fractalkine and platelet-derived growth factor expression was attenuated in U-2 OS cells suggesting that tumor cell-derived 5-LO shapes the tumor microenvironment. In line with the gene expression data, KO of 5-LO had an impact on cell proliferation, motility and MCTS formation. Interestingly, pharmacological inhibition of 5-LO only partly mimicked the KO suggesting that also noncanonical functions are involved.

## Introduction

A number of solid malignancies express arachidonate 5-lipoxygenase (5-LO) although in healthy individuals expression of this enzyme is restricted to leukocytes. To date, it is not known what triggers 5-LO expression in these tumors. Furthermore, the functional consequences on 5-LO expression in tumors are only poorly understood.

5-LO is the central enzyme in the biosynthesis of leukotrienes (LT), 5-HETE and 5-oxo-ETE potent chemotactic lipid mediators that shape the immune response upon an acute inflammation [1]. 5-LO is either located in the cell’s cytosol or in a soluble nuclear compartment. Upon activation the enzyme translocates to the nuclear envelope in a calcium-dependent manner where it accepts arachidonic acid (ARA) from the 5-LO activating protein (FLAP). This lipid has been liberated from membrane phospholipids by a phospholipase such as cytosolic phospholipase A_2α_ (cPLA_2α_) in advance. In the next step, 5-LO converts ARA to 5-HpETE and then further to leukotriene A_4_ (LTA_4_) by insertion of molecular oxygen. 5-LO-derived LTA_4_ is then further converted by the downstream synthases LTA_4_ hydrolase (LTA_4_H) or LTC_4_ synthase (LTC_4_S) to LTB_4_ and the cysteinyl LTs, respectively [1, 2]. Alternatively, 5-LO-derived 5-HpETE can be rapidly converted to 5-HETE by cellular peroxidases followed by further oxidation to 5-oxo-ETE.

Due to their potent pro-inflammatory and immune modulatory functions, LTs and with them 5-LO have been implicated in the pathogenesis of many inflammatory disorders among them asthma, psoriasis, atherosclerosis and rheumatoid arthritis [2]. Furthermore, 5-LO and its products are known to be involved in hematologic malignancies such as acute and chronic myeloid leukemia where they influence cancer stem cell maintenance and confer resistance to tyrosine kinase inhibitors [3–6]. In contrast to leukocytes, other tissues of the human body do not express 5-LO under physiological conditions but substantial expression of the enzyme has been repeatedly found in solid malignancies of the colon [7–10], esophagus [11–13], central nervous system [14], prostate [15, 16], breast [17–19], lung [20], liver [21] and bladder [22]. Furthermore, overexpression of other proteins involved in LT formation and signaling such as cPLA_2α_, FLAP, LTA_4_H, LTC_4_S and the LT receptors has been found in these cancers [23–27]. Expression of 5-LO positively correlates with tumor stage, size, microvessel density and metastatic potential and reduces the responsiveness to cytostatic therapies [8, 10]. Furthermore, 5-LO expressing tumors show a poor prognosis and reduce the overall patient survival [11, 17]. Supporting these findings, 5-LO products have been shown to promote cancer cell proliferation, genotoxicity and activate anti-apoptotic signaling pathways when added to cancer cells in vitro [28–33]. Furthermore, they promote tumor angiogenesis and metastasis in vivo [34].

Although in vitro experimentation employing inhibitors of LT formation and signaling induced cell-cycle arrest and apoptosis in 5-LO expressing cancer cells [35–42], this treatment option has not made it to the clinic yet. Of note, the concentrations used in in vitro studies often exceed the IC_50_ values for inhibition of LT formation/signaling by far. Indeed, it has been recently shown that LT inhibitors such as zileuton, MK-886 and the cysLT1 receptor antagonists interfere with the formation of other lipid mediators such as prostaglandins and epoxyeicosatrienoic acids, activate nuclear receptors and attenuate cell viability independent from 5-LO in concentrations close to their LT inhibiting activity [43–50]. It is therefore hardly possible to deduce a share of LT signaling in carcinogenesis from these pharmacological studies.

A small number of studies used antisense technologies (knock-down) to investigate the role of 5-LO in the carcinogenesis of solid tissues instead of pharmacological inhibition. These reports imply that attenuation of 5-LO expression can negatively influence tumor cell proliferation and viability [33, 49, 51–55]. Still, it is not known what triggers 5-LO expression in solid cancer and in which way the enzyme influences tumor cell function. Is it the enzymatic activity of 5-LO or rather one of the recently found noncanonical functions of the enzyme such as the influence on Wnt/β-catenin signaling, p53 or miRNA processing that gives an advantage to the expressing cells [56]? To answer these questions, we have chosen three different cell lines that express 5-LO, established a complete knockout (KO) of the enzyme in these cells employing CRISPR/Cas-editing and studied the consequences on global gene expression and tumor cell functions.

## Materials and methods

### Materials

PBS, DMEM, McCoy’s 5 A medium, Opti-MEM™ I, sodium pyruvate, penicillin streptomycin concentrate, trypsin-EDTA solution (TE) and StemPro™ Accutase™ were purchased from Gibco Life Technologies (Waltham, MA, USA). Fetal bovine serum (FBS) was obtained from Capricorn Scientific GmbH (Ebsdorfergrund, Germany). Tris, acrylamide 30% (37.5:1), Tween20, Triton-X-100, NP-40 (IGEPAL CA-630) and SDS were bought from PanReac AppliChem ITW Reagents (Darmstadt, Germany). NaCl was purchased from Carl Roth (Karlsruhe, Germany) and EDTA (TitriplexⓇ III) from Merck KGaA (Darmstadt, Germany). Primary antibodies: 5-LO (Abcam^®^, ab169755, Cambridge, UK; Proteintech, #66326, Manchester, UK; 6A12, produced in house), FLAP (Abcam^®^, ab53536), cPLA_2α_ (Santa Cruz Biotechnology^®^, sc-1724, Dallas, TX, USA), LTA_4_H (Santa Cruz Biotechnology^®^, sc-390567) and β-actin (Santa Cruz Biotechnology^®^, sc-8432/sc-1616). Fluorescence-conjugated secondary antibodies (LI-COR^®^ Biosciences, Lincoln, NE, USA).

### Cell lines and transfection

HT-29 (ACC 299) and HCT-116 (ACC 581) cells (DSMZ, Braunschweig, Germany) were maintained in McCoy’s5A and DMEM medium, respectively. U-2 OS cells (ATCC, Manassas, VA, USA) were kept in DMEM. All cell lines were tested for mycoplasma contamination on a regular basis. Media received 10% FBS, 1% sodium pyruvate and 1% penicillin/streptomycin concentrate. For 5-LO KO, cells were transiently transfected with the plasmid ‘lentiCRISPRv2’ (#52961, Addgene, Watertown, MA, USA) carrying a gRNA directed either to exon 2 (target sequence TGGATCACCGGCGATGTCGagg; HCT-116 F5, G6, H11; HT-29 F4; U-2 OS C4) or exon 6 (target sequence TGCAGCGCCGGATCAACACagg; HT-29 A2, G6; U-2 OS C4, H5) of the ALOX5 gene using Lipofectamine^TM^ LTX Reagent with PLUS^TM^ Reagent (Thermo Fisher^TM^). After 72 h cells underwent puromycin selection and limiting dilution. Control cell lines received the empty ‘lentiCRISPRv2’ plasmid and were used as mixed clones.

### Cell lysis and immunoblotting

Cells were lysed in lysis buffer (20 mM Tris-HCl, pH 7.4, 150 mM NaCl, 2 mM EDTA, 1% Triton-X-100, 0.5% NP-40) plus protease and phosphatase inhibitors (cOmplete™ Mini, EDTA-free; PhosSTOP^TM^; Roche Diagnostik GmbH, Mannheim, Germany). Lysates were freeze-thawed, ultrasound homogenized and underwent centrifugation (10,000 rpm, 10 min, 4 °C). Protein concentrations were determined using the Pierce BCA Assay Kit (Thermo Fisher^TM^, Waltham, MA, USA). Equal concentrations of the lysates were separated by SDS-PAGE and transferred to nitrocellulose membranes (LI-COR^®^ Biosciences). PageRuler™ Plus Prestained Protein Ladder (Thermo Fisher^TM^) and r5-LO served as control. After blocking (BIO-RAD Laboratories Inc., Hercules, CA, USA) membranes were scanned with the Odyssey Infrared Imaging System (LI-COR^®^ Biosciences).

### Confocal microscopy

Cells were seeded in 8-well Nunc™ Lab-Tek™ II CC2™ chamber slides (Thermo Fisher^TM^). After 24 h, cells were fixed (4% PFA in PBS), blocked and permeabilized (1% BSA, 0.2% Triton-X-100 in PBS) followed by incubation with the primary antibodies. For detection, a secondary antibody coupled to Alexa FluorR 647 (5-LO) (Thermo Fisher^TM^) was used and nuclei were stained with DAPI. The samples were covered with mounting solution (mowiol + 0.1% (w/v) DABCO), sealed with cover slips and confocal images were acquired (Leica TCS-SP5, Leica Microsystems, Wetzlar, Germany) under identical conditions for pinhole opening, laser power, photomultiplier tension and layer number. During data elaboration by LAS X software (Leica Microsystems), identical parameters were applied for all samples.

### RNA isolation and real-time RT-PCR

RNA isolation was performed using TRIzol^®^ reagent (Thermo Fisher^TM^) according to the manufacturer’s protocol. RNA concentration was determined using a NanoDrop^TM^ 2000 spectrophotometer (Thermo Fisher^TM^) and DNA contaminations were digested using the DNase I RNase-free Kit (Thermo Fisher^TM^). cDNA was produced with the High-Capacity RNA-to-cDNA^TM^ Kit (Thermo Fisher^TM^) according to the manufacturer’s protocol. For RT-PCR experiments, 50 ng cDNA were used as template and PCR products were separated by agarose gel electrophoresis.

RT-qPCR was performed on a StepOnePlus™ Real-Time PCR System (Thermo Fisher^TM^) with PowerUp^TM^ SYBR^TM^ Green fluorescent dye (Thermo Fisher^TM^). Data were normalized to *ACTB* and the respective controls using the 2^−ΔΔCt^ method. Primer sequences can be found in supplementary table 1.

### Lipid mediator formation assay and LC-MS analysis

Lipid mediator formation from 10^7^ cells/ml in ice-cold PGC buffer (PBS, 1 mg/ml glucose, 1 mM CaCl_2_) was initiated by addition of 20 µM ARA plus 2.5 µM Ca^2+^ ionophore A23187. For activity assays with cell homogenates, 10^7^ cells/ml were sonicated (Sonopuls HD 200, sonotrode MS72, BANDELIN electronic GmbH & Co, Berlin, Germany) in ice-cold PBS/EDTA (1 mM) at 4 °C. To prepare 100,000 g supernatants (S100), cell homogenates were subjected to ultracentrifugation (100,000 g, 70 min, 4 °C). Homogenates or S100 were supplemented with ATP (1 mM) and CaCl_2_ (2 mM), pre-heated for 30 sec (37 °C) and lipid mediator formation was initiated by addition of 20 µM ARA. After 10 min the reaction was terminated by addition of 1 ml ice-cold methanol. PMNL which served as control were isolated from leukocyte concentrates (Institut für Transfusionsmedizin und Immunhämatologie, DRK-Bluspendedienst, Frankfurt, Germany) from blood that was drawn with the informed consent of the patients by density gradient centrifugation. Lipid mediators were then analyzed using liquid chromatography tandem-mass spectroscopy (LC-MS/MS) with a 5500 QTrap mass spectrometer (SCIEX, Darmstadt, Germany) operating in negative ESI mode coupled to an Agilent 1200 HPLC system (Agilent Technologies, Santa Clara, CA, USA) and a HTC Pal autosampler (Chrom Tech^®^, Idstein, Germany). Lipid mediators were liquid-liquid extracted (100 µL sample mixed with 100 µL PBS, 20 µL methanol and 20 µL internal standard) twice with 600 µL ethyl acetate. The organic phase was removed at 45 °C under a gentle stream of nitrogen, residues were reconstituted in 50 µL of methanol:water:BHT (50:50:10^–4^, v/v/v) and injected into the LC-MS/MS system. Samples for calibration curve and quality control were prepared similarly. Working solutions of all analytes were prepared in methanol containing 0.1% BHT. The calibration standards were prepared by further dilution of the working standards. Chromatographic parameters: Gemini NX C18 column (150 mm × 2 mm ID, 5 µm, Phenomenex Inc., Torrance, CA, USA); precolumn of the same material; linear gradient (flow rate: 0.5 mL/min; total run time:17.5 min); mobile phases: A water:ammonia (100:0.05, v/v), B acetonitrile:ammonia (100:0.05, v/v). The gradient started at 85% A, changed to 10% A within 12 min, was held for 1 min, shifted back to 85% A in 0.5 min following 3.5 min equilibration. Data were acquired using Analyst software v1.6.2 and quantitation was performed by MultiQuant software v3.0 (SCIEX) using the internal standard method (isotope-dilution mass spectrometry). Calibration curves were calculated by linear regression with 1/x weighting.

### RNAseq

Total mRNA from three biological replicates per sample was isolated using the RNeasy Plus Kit (QIAGEN GmbH, Hilden, Germany) followed by RNA/DNA quality control (Agilent RNA 6000 Nano Kit, Agilent Technologies) according to the manufacturer’s protocols. Library preparation and amplification were performed with the QuantSeq 3'mRNA-Seq Library Prep Kit FWD for Illumina with Unique Dual Indices (Lexogen GmbH, Vienna, Austria) to generate an Illumina compatible library of sequences close to the 3' end of polyadenylated RNA. Library generation was started with oligo dT primers containing the Illumina-specific Read 2 linker sequence. After RNA removal, second strand synthesis was initiated by random priming with primers containing the Illumina-specific Read 1 linker sequence. DNA was then purified and libraries were amplified introducing sequences for library clustering. DNA concentrations were measured with the Qubit 4 fluorometer (Thermo Fisher^TM^). All libraries were mixed in an equimolar fashion according to the average library size and library concentration, diluted to a final concentration of 750 pM and sequenced using a NextSeq 2000 P3 (100 cycle) kit on a Illumina NextSeq 2000 Sequencing System (Illumina Inc., San Diego, CA, USA). Differential expression analysis (DEA) was performed with the Lexogen Quantseq Bluebee platform (trimming: bbduk v35.92; alignment: STAR v2.5.2a; gene read counting: HTSeq-count v0.6.0; differential analysis: DESeq2 pipeline; human genome used for the alignment: Hg38). Genes with a log2-fold change > 1 and adjusted *p*-value < 0.05 were considered to be differentially expressed. Subsequent gene set enrichment analysis was performed employing the NCATs BioPlanet 2019 and GSEAs MSigDB Hallmark 2020 tools via Enrichr [57–59].

### Detection of cytokine release via Enzyme-linked Immunosorbent Assay (ELISA) or cytometric bead array (CBA) method

Secretion of TGFβ_2_ from HCT-116 cells was assessed in the supernatants of cells grown in monolayers for 72 h (24-well plates, 3 × 10^5^ cells/well) in a total volume of 2 mL medium (37 °C, 5% CO_2_, humidified atmosphere). After this, cell supernatants were harvested and cell debris was removed. For TGFβ_2_ measurements, 200 µL of the purified supernatant were acid-activated (40 µL 1 M HCl, 10 min). The samples were then neutralized by addition of 40 µL 1 M NaOH and 200 µL of each sample were measured with the Human TGFB2 ELISA Kit (Invitrogen, Thermo Fisher^TM^) following the manufacturer’s protocol.

For PDGF-AA, fractalkine and MCP-1 release from U-2 OS cells, the cells were cultivated in monolayers (10^5^ cells/well, 24-well plate) for three 3 days (37 °C, 5% CO_2_, in a humidified atmosphere). Subsequently, the medium was changed to 1 ml FCS-free medium and the cells were further incubated for 24 h. After this, 500 µL of the supernatants were harvested and the cell debris was removed. PDGF-AA was measured with the DuoSet^®^ Human PDGF-AA ELISA Kit (R&D systems) following the manufacturer’s protocol. Fractalkine and MCP-1 release was determined employing a bead-based immunoassay (cytometric bead array—CBA, BD Biosciences, Heidelberg, Germany) on a BD FACSVerse™ instrument (BD Biosciences). The assay was performed according to the manufacturer’s protocol using human fractalkine and MCP-1 Flex Sets. Analysis of the FACS data was performed with FCAP Array Software v3.0 (BD™ Biosciences).

### Cell proliferation, viability and colony formation assays

Cell proliferation (starting density: 10^5^ cells/well, 24-well plate) was assessed daily for 4 days. At the end of each time point, cells were detached, stained with trypan blue solution and counted. The capacity to establish single cell colonies in a 2-dimensional environment was measured by seeding 400 cells/well (6-well plate). After 7 days, the cells were fixed with methanol and the colonies formed were stained with Ponceau red and counted. For 3-dimensional colony formation, a single cell suspension in growth medium containing 0.3% agarose (UltraPure^TM^ Agarose, Thermo Fisher^TM^) was plated on top of a 0.5% agarose layer in 12-well-plates. After 3 weeks, colonies were stained with crystal violet and counted.

### Cell death analysis via flow cytometry

Cells pre-incubated with actinomycin D (5, 10 nM), etoposide (20, 40 µM), or 5-fluorouracil (50 µM) for 48 h were harvested and stained with annexinV/propidium iodide [3.5 µL APC annexinV (BD Biosciences, Franklin Lakes, NJ, USA); 3.5 µL PI ([50 µg/ml] Sigma-Aldrich, St. Louis, MO, USA) in 100 µL annexinV binding buffer (BD Biosciences)]. Afterwards, 400 µL of a binding buffer/PBS mix were added to each tube and samples were measured by flow cytometry (BD FACSVerse™, BD Biosciences). Data were analyzed using the FlowJo software (version 10, BD Biosciences).

### Spheroid assays

For spheroid formation, 10^4^ cells in 100 µl growth medium were seeded in spheroid microplates (96-well, Corning Inc., New York, NY, USA) for 15 days. Spheroid growth was monitored daily with a Zeiss Axio Vert.A1 microscope (Carl Zeiss Microscopy Deutschland GmbH, Oberkochen, Germany) and spheroid diameters were measured (Zen blue software, V2.6, Carl Zeiss Microscopy Deutschland GmbH). The number of cells per spheroid was assessed after 7 days by disaggregating 3 spheroids per sample (StemPro™ Accutase™, Thermo Fisher^TM^) and counting the cells manually with a Bürker chamber. Spheroid cell viability was examined using the CellTiter-Glo^®^ 3D Cell Viability Assay (Promega, Madison, WI, USA) according to the manufacturer’s protocol. For the spheroid outgrowth assay, 4-day old spheroids were embedded in Matrigel^®^ (Corning^®^) covered with growth medium and the growth was monitored daily for 14 (HCT-116), 18 (HT-29) and 19 (U-2 OS) days using a Zeiss Axio Vert.A1 microscope (Carl Zeiss Microscopy Deutschland GmbH). The diameter of the spheroids was measured using the Zen blue software (version 2.6, Carl Zeiss Microscopy Deutschland GmbH).

### Transwell migration and invasion assays

400 µL cell suspension [10^6^ cells/mL, growth medium with reduced FBS (0.5%)] were plated into the upper chamber (ThinCert^®^ Tissue Culture Insert, Greiner Bio-One International GmbH, Kremsmünster, Austria) of a 12-well plate while the bottom chamber was filled with 1.2 mL complete growth medium (10% FBS). After 3 h (U-2 OS) or 24 h (HCT-116, HT-29), migrated cells were stained with Calcein-AM (8 µM) in growth medium with reduced FBS (0.5%). Cells adhering to the bottom of the culture insert were detached and fluorescence of the cell suspension was measured (485 nm/520 nm) using an InfiniteR M200 plate reader (Tecan). For invasion the membrane inserts were covered with a thin layer of Matrigel^®^ (Corning^®^) prior to the addition of the cells.

### Statistical analysis

All data are presented as mean + SD. Statistical analysis was performed with GraphPad Prism8 (San Diego, CA, USA). Data were subjected to unpaired *t*-test with Welch’s correction or one-way ANOVA coupled to Dunnett’s post-test for multiple comparisons. No samples were excluded from the analyses.

## Results

### HCT-116, HT-29, Capan-2 and U-2 OS cells express the complete LT biosynthesis machinery but lipid mediator formation is low

**Fig. 1:**
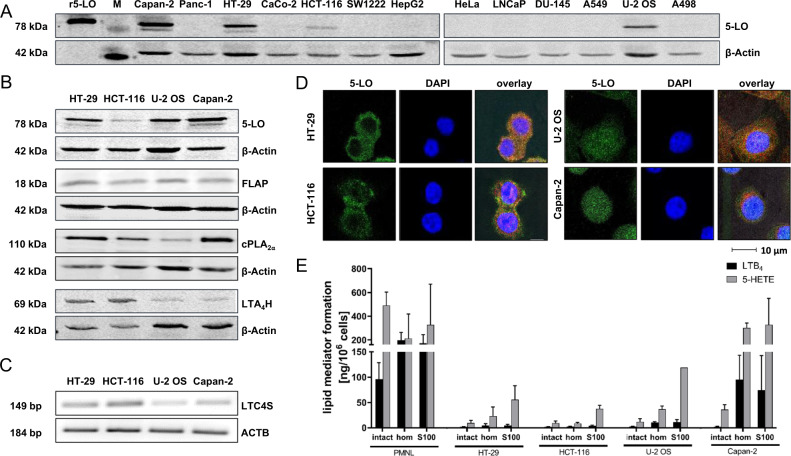
HCT-116, HT-29, Capan-2 and U-2 OS cells express the complete LT biosynthesis machinery but lipid mediator formation is low. **A** Western Blots showing the 5-LO protein expression in several tumor cell lines derived from solid malignancies. **B** Protein expression of 5-LO, FLAP, cPLA_2α_ and LTA_4_ hydrolase in HCT-116, HT-29, U-2 OS and Capan-2 cells. **C** mRNA expression of LTC_4_ synthase (*LTC4S*) in the four cell lines. **D** Confocal microscopy images showing the cellular localization of 5-LO in HT-29, HCT-116, U-2 OS and Capan-2 cells. **E** Comparison of LTB_4_ and 5-HETE formation in intact cells, cell homogenates and 100,000 g supernatants (S100) of human PMNL, HT-29, HCT-116, U-2 OS and Capan-2 cells. The cells were incubated in PGC buffer supplemented with 20 µM ARA and 1 mM Ca^2+^. For formation of 5-LO products the intact cells were stimulated with Ca^2+^ ionophore (A23187, 2.5 μM). The broken cell preparations received 1 mM ATP instead. The samples were then incubated for 10 min at 37 °C and lipid mediator formation was analyzed by LC/MS-MS. The values represent the mean + SD of 3–11 independent experiments. Intact, intact cells; hom, cell homogenates; M, size marker; r5-LO, recombinant human 5-LO; S100, 100,000 g supernatants.

We investigated 5-LO expression in a number of human cell lines derived from solid malignancies (Fig. 1A). From this screening four cell lines with robust 5-LO expression were chosen for further experimentation: The colorectal cancer cell lines HCT-116 resembling colon crypt stem cells (Sadanandam A, 2013 May) and HT-29 displaying colon crypt goblet cell properties, the osteosarcoma cell line U-2 OS and Capan-2 pancreas adenocarcinoma cells. Then, expression of other proteins involved in the biosynthesis of LTs such as cPLA_2α_ important for ARA liberation from cell membranes, the 5-LO down-stream LT synthases LTA_4_H and LTC_4_S as well as FLAP were studied. Each cell line expressed the complete LT biosynthesis machinery (Fig. 1B, C). cPLA_2α_ was highly expressed in HT-29 and Capan-2 cells and to a lower extent in HCT-116 and U-2 OS cells. FLAP and LTC_4_ synthase expression did not substantially differ between the cell lines while LTA_4_H expression was higher in HCT-116 and HT-29 cells (Fig. 1B).

Since all enzymes necessary for LT formation were present in HCT-116, HT-29, U-2 OS and Capan-2 cells, lipid mediator release into the cell culture supernatants was assessed. Surprisingly, the cell lines did not release any 5-LO products during cell culture. Therefore, LT formation upon stimulation with 2.5 µM Ca^2+^ ionophore (A23187) in presence of 20 µM ARA was studied in PGC buffer. Under these conditions, the stimulated tumor cells released LTB_4_ and 5-HETE (Fig. 1E) but only in minute amounts compared to human PMNL.

5-LO activity can be controlled by a number of factors in intact cells among them intracellular localization, phosphorylation, substrate availability and the cellular redox tonus. Therefore, intracellular localization of 5-LO was investigated using confocal microscopy (Fig. 1D). Interestingly, the enzyme showed different localization patterns in the four cell lines. In HT-29 and HCT-116 cells 5-LO was mainly found in the cytosolic compartment while U-2 OS and Capan-2 cells showed an even distribution of 5-LO between cytosolic and nuclear compartments. The inhibition of 5-LO activity by intracellular factors usually can be overcome by disruption of cell integrity as long as Ca^2+^ and ATP are present and the fatty acid substrate is supplemented. Therefore, 5-LO activity was assessed in homogenates and the corresponding 100,000 g supernatants of the cancer cell lines. Disruption of cell integrity elevated 5-LO activity only moderately in HCT-116, HT-29 and U-2 OS cells whereas broken Capan-2 cell preparations displayed a 5-LO product formation comparable to human PMNL (Fig. 1E). These data clearly show that the cancer cells tightly control 5-LO activity keeping lipid mediator formation low during conventional cell culture. The underlying mechanisms are unknown so far and seem to be cell type-dependent.

### Knockout of 5-LO in U-2 OS, HT-29 and HCT-116 cells

**Fig. 2:**
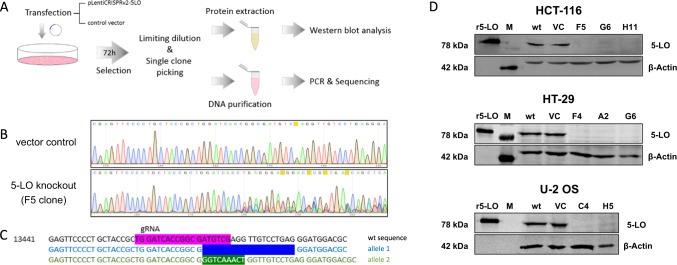
Generation and validation of the 5-lipoxygenase knockout cell lines. **A** Workflow for generation of the 5-LO knockout cell lines. To validate the KO, clones were subjected to Sanger sequencing and compared to the vector control. **B** Depicted here are the sequencing data of the HCT-116 clone F5 and **C** the resulting gene alterations on both alleles compared to the wildtype sequence. The gRNA binding site is marked in pink. Allele 1: Deletion of 19 bases (dark blue); Allele 2: Substitution of 7 bases (ATGTCGA) by nine different bases (GGTCAAACT) (green). The complete sequencing data for all KO clones used in this study can be found in the supplementary information (Supplementary Figs. 1–3). **D** Validation of the 5-LO KO on protein level in HCT-116, HT-29 and U-2 OS single cell clones analyzed by Western blotting. Recombinant human 5-LO was used as positive control. VC empty vector control, M marker, r5-LO recombinant human 5-LO, wt wild-type cells.

The poor activity of 5-LO in the intact cancer cells was surprising and raised the question whether 5-LO’s influence on carcinogenesis is driven by its enzymatic activity or rather by noncanonical functions. To study these aspects, we aimed to eliminate the enzyme from the cancer cell lines using CRISPR/Cas-editing. In addition, empty vector controls were generated for each cell line. Figure 2A depicts the KO procedure followed in this study. Unfortunately, the 5-LO KO was not successful in Capan-2 cells. Therefore, these cells were excluded from further experimentation.

The successful KO of 5-LO in HCT-116, HT-29 and U-2 OS cells was then confirmed by Western Blotting (Fig. 2D). In addition, the genomic DNA of each cell clone was sequenced. Two (U-2 OS C4, H5) or three (HCT-116 F5, G6, H11; HT-29 F4, A2, G6) clones per cell line were chosen for further experimentation. Figure 2B, C illustrate the sequencing data and the resulting gene alterations on both alleles compared to the wildtype sequence for the HCT-116 clone F5. The sequencing data for all HCT-116, HT-29 and U-2 OS KO clones used in this study can be found in the supplementary information (Supplementary Figs. 1–3).

### 5-LO influences the gene expression of U-2 OS, HT-29 and HCT-116 cells

**Fig. 3:**
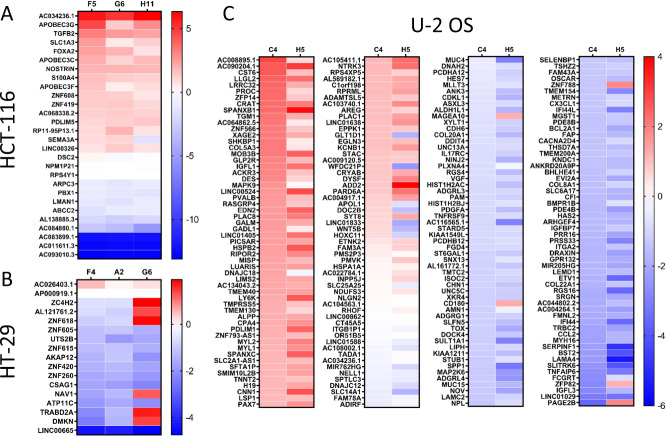
Genome-wide RNA sequencing of the differentially regulated genes after 5-LO KO. Each KO clone and the corresponding empty vector control were measured in three biological replicates during genome-wide RNA sequencing. Genes showing a log2-fold change > 1 and an adjusted *p*-value < 0.05 compared to the empty vector control in each KO clone of a respective cell line were considered differentially expressed. The resulting means for each clone are depicted in heat maps: (**A**) HCT-116, (**B**) HT-29, (**C**) U-2 OS cells. Upregulated genes compared to control vector cells are marked in red while downregulated genes are marked in blue.

To investigate the influence of 5-LO on global gene expression in the three cancer cell lines, next generation sequencing was performed. For this, mRNA was isolated from the 5-LO KO clones as well as their respective empty vector controls and cDNA libraries were established. These libraries were then sequenced using the illumina flow technique. The resulting data were aligned to the human genome (Hg38) and differential expression analysis (DEA) was performed comparing each KO clone to its corresponding vector control. Only genes that showed significant differential regulation in all KO clones of the same cell line were considered to be influenced by 5-LO and therefore chosen for further investigation. The mean regulation of the genes that were found to be differentially regulated upon 5-LO KO in U-2 OS, HT-29 and HCT-116 cells are presented as heat maps in Fig. 3A–C.

The total number of differentially regulated genes (DEG) was highly variable between the cell lines: In U-2 OS cells a total of 234 DEGs was detected upon 5-LO KO while in HCT-116 and HT-29 cells only 28 and 18 DEGs were found, respectively. In HCT-116 cells about 60% of the genes affected were upregulated upon 5-LO KO and 40% were downregulated while in HT-29 cells the majority of DEGs were downregulated. In U-2 OS cells 90 genes were consistently upregulated and 129 genes were downregulated in both clones. Interestingly, there was no overlap in DEGs between the three cell lines.

To identify possible cellular processes affected by 5-LO in HCT-116, HT-29 and U-2 OS cells, we further analyzed the RNAseq data via Enrichr employing the BioPlanet 2019 and MSigDB Hallmark 2020 platforms (Supplementary Fig. 4). This analysis showed that the 5-LO KO had an impact on proteins involved in cell adhesion processes and extracellular matrix (ECM) formation, G protein signaling and cytoskeleton organization in U-2 OS cells suggesting an influence on epithelial to mesenchymal transition (EMT) and the inflammatory response. Due to the lower number of genes affected in HT-29 and HCT-116 cells Enrichr analysis was less successful but nevertheless suggested an influence on G protein signaling, transmembrane transport and generic transcription for HT-29 cells and an involvement in inflammatory reactions and cytoskeleton organization for HCT-116 cells. In addition to the analyses performed with Enrichr, the EMTome platform [60] as well as a thorough literature search was performed for the DEGs. Table 1 shows a comprehensive overview of this search grouping the DEGs according to their published functions in cellular processes known to be involved in tumorigenesis such as EMT, migration, G protein signaling, adhesion/ECM formation and cytoskeleton organization.

**Table 1:** Comprehensive overview of the differentially expressed genes per cell line grouped according to their involvement in cellular processes important for tumorigenesis. Genes involved in more than one process are listed several times.

	Epithelial to mesenchymal transition		Migration			Extracellular matrix/adhesion	Transcription	G protein signaling	cytoskeleton organization
**HCT-116**	DSC2		ARPC3	PBX1	S100A4	DSC2	APOBEC3C		ARPC3
	FOXA2		DSC2	PDLIM5	SEMA3A	TGFB2	APOBEC3G		S100A4
	PBX1		FOXA2	RPS4Y1	TGFB2	S100A4	ZNF419		TGFB2
	S100A4		NOSTRIN				ZNF608		
	TGFB2								
**HT-29**	AKAP12		AKAP12			DMKN	ZNF260	AKAP12	CSAG1
	DMKN		DMKN				ZNF420		NAV1
	TRABD2A		NAV1				ZNF605		
			TRABD2A				ZNF615		
							ZNF618		
**U-2 OS**	ACKR3	MIR205HG	ACKR3	IFI44	PLAC1	ADAMTSL5	HES7	ADGRG1	ADD2
	ADGRG1	MUC15	ADD2	IFI44L	PLAC8	BST2	MLLT3	ADGRL3	AMN1
	AREG	MUC4	ADGRG1	IGFBP7	PLXNA4	CDH6	TSHZ2	ADGRL4	ANK3
	BCL2A1	NOV	AREG	IL17RC	PRR16	COL20A1	ZFP14	ARHGEF4	ARHGEF4
	BHLHE41	NTRK3	ARHGEF4	ITGA2	RGS16	COL22A1	ZFP82	CHN1	CNN1
	CCL2	PAM	BCL2A1	ITGB1BP1	RGS4	COL5A3	ZNF566	DOCK4	DES
	CDH6	PARD6A	BHLHE41	KCNB1	RHOF	COL8A1	ZNF788	GLP2R	DNAH2
	CHN1	PAX7	BST2	LAMA4	RIPOR2	CST6	ZNF793-AS1	KNDC1	DRAXIN
	CNN1	PDE4B	CCL2	LAMC2	SELENBP1	FAP		OR51B5	EPPK1
	CPA4	PDGFA	CDH6	LEMD1	SERPINF1	HAS2		PARD6A	FGD4
	CRYAB	PLAC1PLAC8	CDKL1	LIPH	SHKBP1	ITGA2		PMVK	FMNL2
	CST6	PRR16	CHN1	LRRC32	SLC2A1-AS1	ITGB1BP1		RASGRP4	KIAA1211
	CX3CL1	RGS16	CNN1	LSP1	SLFN5	LAMA4		RGS16	LLGL2
	DES	RGS4	CRYAB	MAGEA10	SPANXB1	LAMC2		RGS4	LSP1
	DOC2B	RHOF	CST6	MAP2K6	SPANXC	MUC15		RHOF	MISP
	DRAXIN	SELENBP1	CX3CL1	MAPK9	SPP1	MUC4		RIPOR2	MYH16
	ETV1	SERPINF1	DDIT4	METRN	SRGN	NINJ2			MYL1
	FAP	SHKBP1	DES	MGST1	ST6GAL1	NOV			MYL2
	FMNL2	SLC2A1-AS1	DOC2B	MIR205HG	STUB1	PARD6A			PARD6A
	HAS2	SLFN5	DRAXIN	MUC15	SYT8	PCDHA12			PLXNA4
	IGFBP7	SPANXB1	DYSF	MUC4	TADA1	PCDHB12			RHOF
	ITGA2	SPP1	EDN2	MYL2	TGM1	SPP1			SNX13
	LAMA4	ST6GAL1	EGLN3	NDUFS3	THSD7A	SRGN			THSD7A
	LAMC2	STUB1	EPPK1	NELL1	TMEM130	STUB1			TNNT2
	LEMD1	TGM1	ETNK2	NINJ2	TNFAIP6	TGM1			UNC5C
	LIPH	THSD7A	ETV1	NOV	TNFRSF9	TMTC2			
	MAGEA10	TNFAIP6	EVI2A	NTRK3	TOX	TNFAIP6			
	MAP2K6	VGF	FAM3A	PAM	TSHZ2	XYLT1			
	MGST1	WNT5B	FAP	PARD6A	UNC5C				
			FMNL2	PAX7	VGF				
			GADL1	PDE4B	WNT5B				
			GPR132	PDGFA	XKR4				
			HAS2	PICSAR	ZFP82				
			HOXC11						

**Fig. 4:**
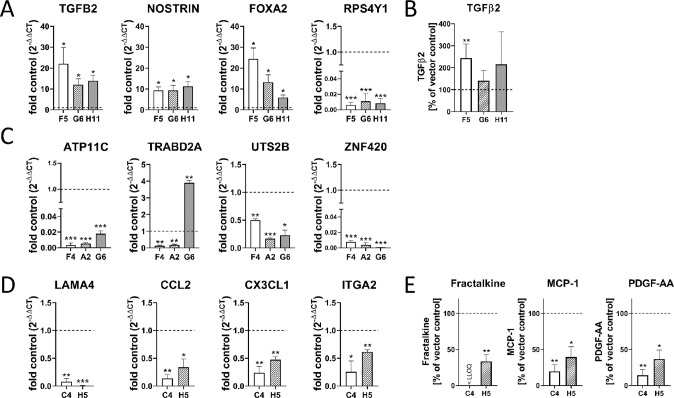
Validation of the RNAseq data via mRNA and protein expression employing RT-qPCR, ELISA and CBA. The DEGs were further validated by RT-qPCR and analysis of protein expression. Four differentially regulated genes per cell line are depicted. Gene expression was normalized to *ACTB* (housekeeping gene) and the corresponding control vector cells (2^−ΔΔct^ method). **A** qPCR analysis of four representative DEGs from HCT-116 5-LO KO cells. **B** Secretion of TGFβ_2_ into the cell culture supernatants of HCT-116 5-LO KO cells measured via ELISA. Depicted are the relative TGFβ_2_ amounts in % compared to the vector control (mean TGFβ_2_ secretion from vector control cells: 231.9 ± 140 pg/µg total protein). **C** qPCR analysis of four representative DEGs from HT-29 5-LO KO cells and **D** U-2 OS cells. **E** Cytokine release from U-2 OS 5-LO KO cells. Data are depicted as relative cytokine amounts in % compared to the corresponding control vector cells. PDGF-AA was assessed by ELISA while fractalkine (*CX3CL1*) and MCP-1 (*CCL2*) were measured via FACS employing a cytometric bead array (mean secretion from vector control cells: fractalkine, 64.9 ± 9.2 pg/µg protein; MCP-1, 978.2 ± 101.8 pg/µg protein; PDGF-AA, 255,6 ± 95.6 pg/µg protein). The complete mRNA expression data can be found in supplementary Fig. 4 and supplementary table 1. All data are presented as mean + SD of three independent experiments. Asterisks indicate significant changes vs. control vector cells. *P ≤ 0.05, **P ≤ 0.01, ***P ≤ 0.001.

Next, the DEGs were validated by qPCR. In HCT-116 cells *TGFB2* (12–22-fold), the transcription factor *FOXA2* (5- to 25-fold) as well as *NOSTRIN* (10-fold) displayed high upregulation upon 5-LO KO on mRNA level while the ribosomal protein *RPS4Y1* was the most potently downregulated gene (120-fold) in these cells (Fig. 4A). Measurement of TGFβ_2_ from the cell culture supernatants of the HCT-116 5-LO KO clones confirmed the upregulation of the gene on protein level (Fig. 4B).

In HT-29 KO cells qPCR measurements confirmed the downregulation of a number of Krüppel-associated box domain (KRAB) zinc finger proteins among them *ZNF420* (125–1300 fold) as well as the phospholipid flippase *ATP11C* (50–300 fold) and *UTS2B* (urotensin 2B; 2–6 fold). In addition, a number of genes showed a downregulation in clones F4 and A2 while their expression was upregulated in clone G6 (e.g. *TRABD2A*) suggesting clonal effects in the latter (Fig. 4C).

In U-2 OS cells a huge number of genes was influenced upon KO of 5-LO among them important chemokines such as *CCL2* (MCP-1) and *CX3CL1* (fractalkine), the mitogen *PDGFA* (platelet-derived growth factor α) as well as various collagens and laminins (Fig. 4D). Suppression of MCP-1, fractalkine and PDGFα expression was additionally confirmed on protein level (Fig. 4E). The complete mRNA expression data for the HT-29 and HCT-116 KO clones can be found in supplementary Fig. 5. The qPCR data for the U-2 OS KO clones can be found in supplementary table 1.

### 5-LO influences cell growth and viability of HT-29, HCT-116 and U-2 OS cells

**Fig. 5:**
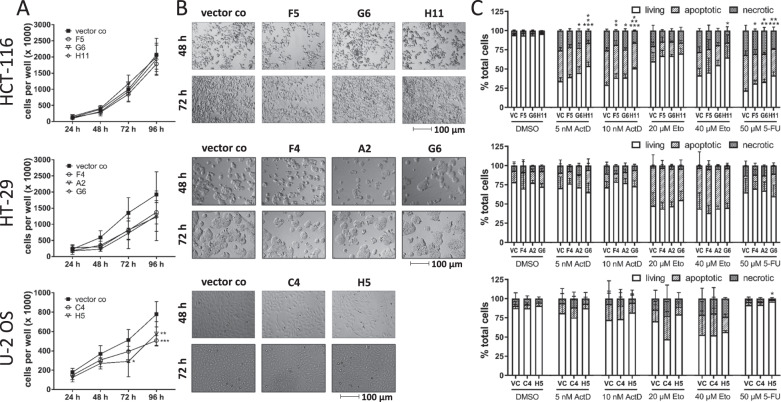
Influence of the 5-LO KO on cell growth and survival of HCT-116, HT-29 and U-2 OS cells. **A** Cell proliferation of the cells was assessed by counting the cells on a daily basis for 4 days. **B** In addition, pictures of the proliferating cells were taken on a daily basis. **C** Cell death analysis of the cells after treatment with actinomycin D (5, 10 nM), etoposide (20, 40 µM) or 5-fluorouracil (50 µM). DMSO treated cells were used as negative control. After 48 h the cells were stained with annexinV and propidium iodide and cell death was analyzed by flow cytometry. Data are presented as mean + SD from three independent experiments. Asterisks indicate significant changes vs. control vector treated cells. *P ≤ 0.05, **P ≤ 0.01, ***P ≤ 0.001. ActD actinomycin D, Eto etoposide, VC empty vector control, 5-FU 5-fluorouracil.

We investigated the growth and viability of the HT-29, HCT-116 and U-2 OS 5-LO KO cells and compared this to the corresponding vector controls. For this, the cells were seeded in 24-well plates and cell numbers were assessed after 24, 48, 72 and 96 h (Fig. 5A). In addition, pictures of the cell layers were taken (Fig. 5B). Absence of 5-LO significantly attenuated HT-29 and U-2 OS cell proliferation while the growth of HCT-116 cells was not affected (Fig. 5A). Furthermore, mitochondrial activity as a measure of cell viability was assessed after 48 h of cell culture employing the WST-1 assay and cell cycle analysis was performed. KO of 5-LO did not substantially influence overall viability in the three cell lines (Supplementary Fig. 6A). Furthermore, cell cycle distribution was not affected by 5-LO (Supplementary Fig. 7).

In the past, 5-LO has been implicated in conferring chemoresistance to overexpressing tumors [61]. Therefore, survival of HCT-116, HT-29 and U-2 OS cells was studied after treatment with cytostatic drugs (actinomycin D, 3.5–14 nM; etoposide, 10–50 nM) for 48 h. The effects of 5-LO on cancer cell viability were weak and clone-dependent (Supplementary Fig. 6B). In contrast, analysis of cell death induction via flow cytometry using annexinV/propidium iodide staining showed that absence of 5-LO significantly elevated the proportion of living cells in HCT-116 5-LO KO clones upon treatment with Actinomycin D. This suggests that 5-LO might be involved in cell death induction in these cells (Fig. 5C). The cell death induced by cytotoxic compounds was not influenced by 5-LO in HT-29 and U-2 OS cells.

### 5-LO influences in vitro tumor cell function of HT-29, HCT-116 and U-2 OS cells

We studied the 5-LO KO cell lines in a number of functional assays that address critical factors involved in tumor establishment and maintenance, tissue invasion and metastasis.

**Fig. 6:**
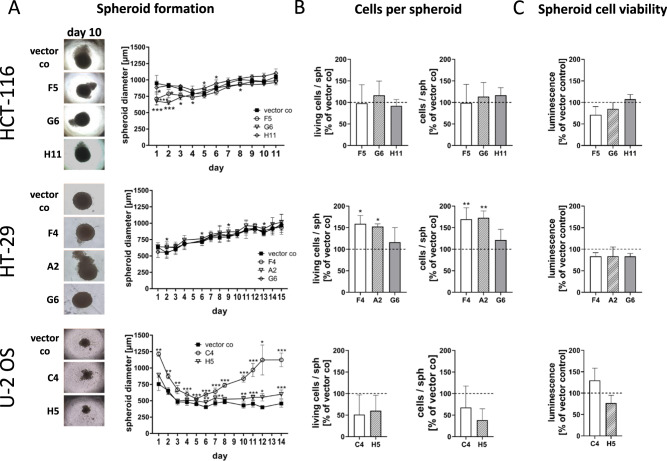
Influence of the 5-LO KO on MCTS formation of HCT-116, HT-29 and U-2 OS cells. **A** Three dimensional growth of the 5-LO KO clones and vector control cells as multicellular tumor spheroids. For this, cells were seeded in low-attachment plates and the spheroid diameter (in µm) was monitored for 11–16 days. Data are presented as mean ± SD of 3–9 independent experiments. **B** Number of living (left graph) and total (right graph) cells per spheroid. Data are presented as mean + SD of three independent experiments. **C** Cell viability in spheroids was assessed by the CellTiter-Glo® 3D cell viability assay. Data are presented as mean + SD of three independent experiments. Data depicted in **B** and **C** are depicted as % vector control. Asterisks indicate significant changes vs. control vector treated cells. *P ≤ 0.05, **P ≤ 0.01, ***P ≤ 0.001.

The capacity to form multicellular tumor spheroids (MCTS) was investigated in low-attachment microplates. HCT-116 cells formed big, dense spheroids (mean diameter at day 7: 924 ± 66 µm). MCTS size stayed constant over time (Fig. 6A) and the 5-LO KO had no influence on MCTS growth and appearance. HT-29-derived MCTS were also dense in appearance, but formed smaller spheroids at the beginning (mean diameter at day 7: 784 ± 47 µm) compared to HCT-116 cells. These spheroids grew in a time-dependent manner. Absence of 5-LO had no influence on the size of the core spheroid in HT-29 cells but led to the outgrowth of large cell clusters from the spheroid at later time points of the incubation (Fig. 6A). In accordance, the number of living cells was elevated in MCTS from HT-29 5-LO KO clones (Fig. 6B). Compared to HCT-116 and HT-29 cells, U-2 OS wild-type cells formed rather small MCTS (mean diameter at day 7: 463 ± 41 µm) with a less dense appearance and blurry outlines. Interestingly, absence of 5-LO strongly attenuated the formation of coherent spheroidal structures from the 5-LO KO clones resulting in loose cell clusters with increased diameter (Fig. 6A). Accordingly, the number of living cells was reduced in these spheroids although not in a significant manner (Fig. 6B). MCTS cell viability measured by ATP consumption was not influenced by 5-LO in any cell line (Fig. 6C).

**Fig. 7:**
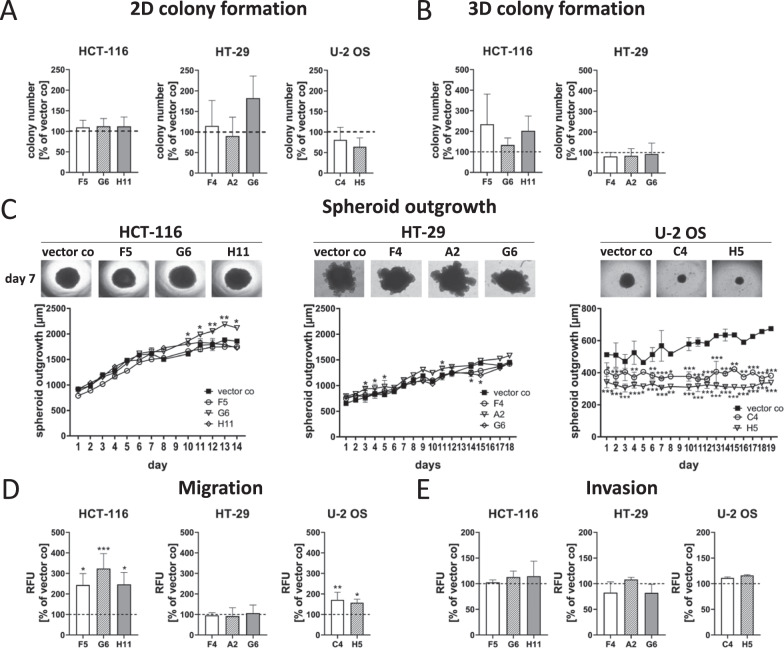
Influence of the 5-LO KO on colony formation, migration and invasion of HCT-116, HT-29 and U-2 OS cells. **A** Two dimensional colony formation of the 5-LO KO clones. Cells were seeded into 6-well plates at low density (400 cells/well) and the number of colonies formed after 7 days was assessed by Ponceau red staining. Data are presented as + SD of three independent experiments (Plating efficiency of control vector cells: HCT-116; 72%, HT-29; 70%, U-2 OS; 80%). **B** Three dimensional colony formation of the 5-LO clones in soft agar. Colonies formed after 3 weeks were stained with crystal violet and counted. U-2 OS cells did not form any colonies under these conditions. Data are presented as mean + SD of three independent experiments (Plating efficiency of control vector cells: HCT-116; 1.6%, HT-29; 0.6%). **C** Outgrowth/invasion of preformed spheroids into matrigel^®^. Growth (diameter in µm) was monitored for 14–19 days. Data are presented as mean ± SD of 3–9 independent experiments. **D** Directed cell migration towards serum was measured in a transwell assay for 3 h (U-2 OS) or 24 h (HCT-116, HT-29). **E** For investigations on invasive properties of the cells migration through matrigel^®^ was measured. Data on cell migration and invasion in the transwell assay are presented as mean + SD from 3–5 independent experiments. Data depicted in **A**, **B**, **D**, and **E** are depicted as % vector control. Asterisks indicate significant changes vs. control vector treated cells. *P ≤ 0.05, **P ≤ 0.01, ***P ≤ 0.001.

Next, the influence on tumor cell clonogenicity and anoikis was assessed using 2D and 3D colony formation assays. The ability to establish colonies in 2D cell culture was investigated by seeding single cell suspensions of the KO cell lines at low density (400 cells per well) in 6-well plates. After 7 days, the colonies formed were fixed and stained, colony numbers were counted and the average colony size was assessed. Absence of 5-LO did not affect colony formation and plating efficiency in HCT-116 and HT-29 cells, while U-2 OS colony numbers were slightly reduced, although not in a significant manner (Fig. 7A).

3D colony formation was monitored by embedding cell suspensions of the 5-LO KO clones in soft agar. After 3 weeks, the established colonies were stained with crystal violet and colony numbers were counted. HCT-116 and HT-29 KO and vector control cells readily formed colonies but 5-LO had no impact on this. Since U-2 OS cells did not establish any colonies in soft agar, assessment of 3D colony formation was not possible (Fig. 7B).

A substantial number of DEGs found in the RNAseq analysis are involved in EMT and/or affect cancer cell migration. Furthermore, a remarkable number of the DEGs is connected to the cell’s cytoskeletal organization, plays a role in cell adhesion and ECM formation (Table 1) suggesting that 5-LO influences cancer cell motility and adhesion. For this reason, a number of assays characterizing the migratory and invasive capacity of the cells were performed. First, haptotaxis was studied as a measure of local cellular outgrowth influenced by cell surface and ECM structures. For this, a wound closure assay was chosen. The cells were seeded in 24-well plates containing inserts partly covering the growth area of the well. Upon cell confluence, the inserts were removed creating a physical gap within the cell monolayer and the closure time of this space was monitored. 5-LO did not influence haptotaxis in the three cell lines (Supplementary Fig. 8).

In addition to haptotaxis, directed migration towards a serum gradient was assessed in a transwell assay. After a defined time (24 h: HCT-116, HT-29; 3 h: U-2 OS), cells that had transmigrated through the porous membrane were stained with calcein-AM, detached and fluorescence intensity of the cell suspensions was measured. Indeed, HCT-116 as well as U-2 OS 5-LO KO clones showed an elevated migratory capacity of 200–300% and 150%, respectively, compared to the corresponding vector controls (Fig. 7D). Migration of HT-29 cells was not influenced by 5-LO.

To study the invasive properties of the cells, we generated MCTS from each cell line by cultivation in low-attachment microplates. After 4 days, the resulting spheroids were then embedded into matrigel. Outgrowth of the spheroids into the surrounding matrix was monitored for 14–19 days and the spheroid diameters were calculated on a daily basis (Fig. 7C). Embedded MCTS from the HCT-116 vector control showed a dense appearance and time-dependent growth. Absence of 5-LO did not influence this behavior. Matrigel embedded HT-29 vector control MCTS readily invaded the surrounding matrix forming complex protrusions with lower density resembling colonic crypts. Again, MCTS outgrowth was not influenced by the KO. In contrast, 5-LO had a strong impact on the growth and invasion of U-2 OS MCTS in matrigel. While embedded vector control-derived MCTS had a dense appearance, grew in a time-dependent manner and invaded the surrounding matrix with tiny tube-like protrusions, growth of spheroids derived from the 5-LO KO clones stagnated over time and the tube-like protrusions were not formed. In addition, directed invasion through matrigel was studied in a transwell assay. In contrast to migration, directed invasion was not influenced by 5-LO in any of the cell lines (Fig. 7E).

### Pharmacological inhibition of 5-LO partially mimics the knockout

**Fig. 8:**
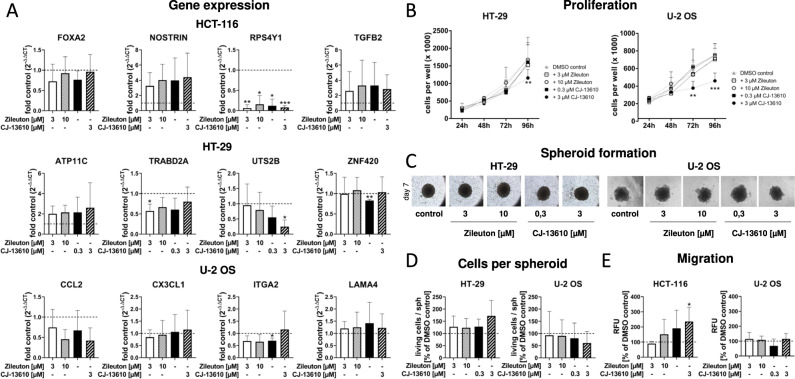
Pharmacological inhibition of 5-LO partially mimics the 5-LO KO. **A** mRNA expression of selected genes in HCT-116, HT-29 and U-2 OS cell expressing wild-type 5-LO after treatment with different 5-LO inhibitors (zileuton (3 or 10 µM)); CJ-13610 (0.3 or 3 µM) for 3 days measured by RT-qPCR. Each gene was normalized to *ACTB* as well as the DMSO treated control (2^-ΔΔct^ method). Data are presented as mean + SD of three independent experiments. **B** Cell proliferation in HT-29 and U-2 OS cells treated with zileuton (3 or 10 µM) or CJ-13610 (0.3 or 3 µM) for 96 h. Data are presented as mean + SD of 3–5 independent experiments. **C** Three dimensional growth of HT-29 and U-2 OS cells treated with zileuton (3 or 10 µM) or CJ-13610 (0.3 or 3 µM) during MCTS formation. **D** Number of living HT-29 and U-2 OS cells per spheroid depicted as % of the corresponding DMSO control. Data are presented as mean + SD of three independent experiments. **E** Directed cell migration of HCT-116 and U-2 OS cells towards serum after treatment with zileuton (3 or 10 µM) or CJ-13610 (0.3 or 3 µM). After 3 h (U-2 OS) or 24 h (HCT-116), migrated cells were stained and fluorescence of the cell suspension was measured. Data are presented as mean ± SD from 3–5 independent experiments. Asterisks indicate significant changes vs. DMSO control. *P ≤ 0.05, **P ≤ 0.01, ***P ≤ 0.001.

Since 5-LO activity was very low in intact HT-29, HCT-116 and U-2 OS cells (Fig. 1E), we postulated that the enzyme’s noncanonical functions might influence cancer cell gene expression and function. To answer this question, U-2 OS, HCT-116 and HT-29 cells carrying the wild-type enzyme were treated with the 5-LO inhibitors zileuton (3 and 10 µM) or CJ-13,610 (0.3 and 3 µM) for 3 days. Then, expression of selected DEGs found in the RNAseq analysis was reassessed by qPCR (Fig. 8A). For HCT-116 cells three upregulated genes (*TGFB2*, *NOSTRIN*, *FOXA2)* and one downregulated gene (*RPS4Y1*) were chosen. *TGFB2, NOSTRIN* and *RPS4Y1* were also regulated by pharmacological inhibition of 5-LO (3 ± 0.3,4 ± 0.4 –fold and 0.1 ± 0.04, respectively). In contrast, the inhibitors had no influence on the expression of *FOXA2*. In HT-29 cells expression of *ATP11C*, *TRABD2A*, *UTS2B* and *ZNF420* was studied after 5-LO inhibitor treatment. All four genes showed potent downregulation in the 5-LO KO cells but 5-LO inhibitor treatment had no influence on *ATP11C*, *TRABD2A* and *ZNF420* expression. Only high concentrations of CJ-13,610 (3 µM) attenuated *UTS2B* levels by about 80%. In U-2 OS cells, *CCL2*, *CX3CL1*, *ITGA2* and *LAMA4* expression was investigated. While *CCL2* expression was partially downregulated by the inhibitors, expression of the other genes was not affected. These inhibitor data clearly show that canonical as well as noncanonical functions of 5-LO seem to control cancer cell gene expression.

Next, the experiments in which the 5-LO KO had shown an effect on cancer cell function were repeated with inhibitor treated wild-type cells. As expected, these assays also pointed to a mixed influence of canonical and noncanonical 5-LO functions. Proliferation of U-2 OS and HT-29 cells was attenuated upon pharmacological 5-LO inhibition but these effects were less pronounced compared to the KO cells and only 3 µM CJ-13,610 showed statistical significance (Fig. 8B). Furthermore, 5-LO inhibition could neither recapitulate the influence on U-2 OS spheroid formation capacity nor the elevated cell numbers and extrusions formed in HT-29 5-LO KO spheroids (Fig. 8C, D). Interestingly, directed migration of HCT-116 cells was also induced upon inhibition of 5-LO while U-2 OS cell migration was not affected by the inhibitors (Fig. 8E).

## Discussion

In contrast to healthy tissues, 5-LO expression is frequently found in solid tumors where it positively correlates with tumor progression. The consequences of the enzyme’s expression in these malignant tissues is only poorly understood. In the present manuscript we studied the impact of 5-LO expression in the four cancer cell lines HCT-116, HT-29, U-2 OS and Capan-2. In our hands the cell lines expressed the complete LT biosynthesis machinery but did not form substantial amounts of bioactive lipids even after stimulation with Ca^2+^ ionophore and supplementation with ARA.

It is known that 5-LO activity can be attenuated by a number of intracellular factors such as the cell’s redox tonus and the intracellular localization of the enzyme [1, 2]. Interestingly, 5-LO localization was different in the four cell lines. While enzyme expression was mainly cytosolic in the two colon carcinoma cell lines, 5-LO was evenly distributed between the nucleus and the cytosol in U-2 OS and Capan-2 cells. It is known that PKA-dependent phosphorylation of Ser523 situated in the nuclear import sequence of 5-LO is sequestering the enzyme in the cytosol and also attenuates its activity [62, 63]. This might explain the low activity in the cancer cells. Unfortunately, we were not able to explore this issue further since all commercially available anti-phosphoSer523 antibodies that we used were unspecific in our hands.

Some of the factors that impair 5-LO activity in intact cells can be overcome when cellular integrity is disrupted. Indeed, we found that 5-HETE and LTB_4_ formation was higher in the homogenates of all four cancer cell lines and the corresponding 100.000 g supernatants. Nevertheless, 5-LO activity in HT-29, HCT-116 and U-2 OS cells was very low compared to human PMNL. In contrast, activity in S100 preparations of Capan-2 cells almost equalled PMNL activity. Our data clearly show that 5-LO expressing cancer cells suppress the enzyme’s activity in conventional cell culture. Judged by the cellular localization patterns of 5-LO and its different activities in broken cell preparations, this suppression seems to be cell type-dependent. The mechanisms by which 5-LO activity is controlled in cancer cells is not known so far and therefore further investigations are needed in the future to answer this important aspect.

It is not surprising that 5-LO activity is tightly controlled in cancer cells since biosynthesis of lipid mediators is accompanied by the accumulation of highly reactive lipid species. These lipids can severely damage DNA and RNA structures and also trigger apoptotic and ferroptotic cell death programs [61, 64–69]. Furthermore, 5-LO products such as LTB_4_ and 5-oxo-ETE can draw unwanted attention to the tumor due to their potent chemoattractant properties on neutrophils and monocytes. It can be speculated that 5-LO expression and activity in cancer cells is subject to environmental changes thereby adjusting enzyme expression to current needs. Indeed, unpublished data from our group show that cancer cells can upregulate 5-LO expression and activity upon cell stress.

So the question is, why do tumors still take the trouble of expressing this enzyme? Which benefits might come with it and is it the enzymatic activity of 5-LO that gives an advantage to the cells or do noncanonical functions such as the influence on Wnt signaling and miRNA processing play a role [56]?

In the past, a number of reports employing pharmacological tools such as 5-LO and FLAP inhibitors as well as LT receptor antagonists have shown to have an impact on cancer cell proliferation. These studies have to be treated with care since a majority of the inhibitors used are known to have off-target activities with potencies comparable to LT inhibition. Among these are the attenuation of prostaglandin and epoxyeicosatrienoic acid formation as well as the activation of PPARγ. Furthermore, the compounds were shown to attenuate cell viability independent from 5-LO [43–50]. It is therefore not possible to draw any conclusions on the share of LT signaling in carcinogenesis from these publications.

Instead of pharmacological inhibition, a number of studies has used antisense technologies to attenuate 5-LO activity. These data show that 5-LO can influence tumor cell proliferation and viability [33, 49, 51–55]. Curiously, cellular functions such as migration, invasion and establishment of multicellular tumor spheroids have never been investigated in these studies. Since antisense technologies are only able to knock-down the protein of interest residual low expression of this protein remains which might be still sufficient to maintain certain cellular functions. In order to avoid this, 5-LO was knocked-out from the cancer cell lines with the help of CRISPR/Cas-editing in the present study. To our knowledge, this is the first time that this has been done in human cancer cells. Then, the influence on cancer cell gene expression and cellular functions was investigated.

Interestingly, the genes we found to be differentially regulated in the RNAseq analysis did not overlap between HT-29, HCT-116 and U-2 OS cells. The three cell lines substantially differ in terms of origin and differentiation (HCT-116 - colon crypt stem cell-like cells; HT-29 – colon crypt goblet cell-like cells; U-2 OS—osteosarcoma fibroblast-like cells) resulting in diverging protein expression patterns. U-2 OS cells for example release high amounts of MCP-1 while HT-29 and HCT-116 cells do not express this cytokine. It is therefore not particularly surprising, that there was no overlap in the genes affected by the 5-LO KO.

A number of DEGs that were found in the RNAseq analysis have already been described to be influenced by 5-LO and its products among them the cytokines *TGFB2* (TGFβ_2_), *CCL2* (MCP-1), *CX3CL1* (fractalkine) and *PDGFA* (platelet-derived growth factor, subunit α) as well as collagens, laminins and semaphorin-3A. TGFβ is a central regulator of cancer progression, EMT and fibrosis [70]. We found that TGFβ_2_ as well as the TGFβ activator *LRRC32* are upregulated upon 5-LO KO in HCT-116 and U-2 OS cells, respectively. In accordance, 5-LO has been found to suppress TGFβ in the lungs of mice inoculated with Schistosoma mansoni eggs [71]. MCP-1, fractalkine and PDGF are cytokines which are involved in tumor cell proliferation, migration and metastasis as well as the recruitment and differentiation of tumor-associated macrophages and fibrocytes [72–74]. In the present study, expression of these cytokines positively correlated with 5-LO expression in U-2 OS cells. Indeed, expression of these cytokines has already been linked to 5-LO. MCP-1 expression is positively correlated with 5-LO activity in human vascular smooth muscle cells, synovial fibroblasts and in a mouse model of hepatic inflammation [75–77]. CX3CL1 has been found downregulated in the cortex of 5-LO^-/-^ mice and LTB_4_ is known to stimulate PDGF release from rat aortic smooth muscle cells [78, 79]. In addition to cytokines, expression of proteins involved in cell adhesion and migration such as collagens (*COL5A3*, *COL8A1*, *COL20A1*, *COL22A1*), laminins and laminin regulators (*LAMA4*, *LAMC2*, *DRAXIN*) were attenuated in U-2 OS cells upon 5-LO KO. Altraja et al. recently found that laminin β2 is upregulated by LTD_4_ in human bronchial epithelial cells [80]. Furthermore, 5-LO and LTB_4_ induce expression of collagens in murine macrophages and in a mouse model of eosinophil-mediated respiratory inflammation [81, 82]. *SEMA3A*, a protein involved in axonal growth and angiogenesis, has already been found upregulated in the brains of mice treated with the 5-LO inhibitor zileuton [83].

Pathway analysis of the RNAseq data showed that although there is no overlap in DEGs, the cellular processes affected by 5-LO such as EMT, ECM formation, cell adhesion, migration, GPCR signaling, cytoskeleton organization and gene transcription were partly congruent. This suggests that 5-LO is involved in cell motility, tumor establishment, chronic inflammation and fibrosis.

The functional consequences of the 5-LO KO did correspond to the pathways found in the gene set enrichment analysis. Knock-out of 5-LO significantly attenuated the proliferation of HT-29 and U-2 OS cells. This was only partly dependent on 5-LO activity since the 5-LO inhibitor CJ-13,610 showed a comparable effect. These data are in accordance with a number of studies showing that knock-down of 5-LO attenuates cell proliferation and viability [33, 49, 51–55].

It has been shown in the past that presence of 5-LO confers resistance to cytostatic drug-induced cell death [61]. We found that absence of 5-LO attenuated the viability of U-2 OS cells upon actinomycin D treatment. In contrast, the proportion of living HCT-116 cells was elevated under actinomycin D and 5-FU treatment in the KO clones. These data suggest that presence of 5-LO can either support cell survival or cell death under cell stress conditions and that the outcome on cellular viability is dependent on the cell type. It also underlines that presence of 5-LO can be detrimental to cells under stress conditions. In line with this, 5-LO products have been recently implicated in ferroptosis induction [84, 85]. Furthermore, 5-LO is a known p53 target gene [86]. This strongly suggests that the enzyme has a role in cell death induction and explains the tight control of its enzymatic activity in the tumor cells.

But why do tumors still overexpress this enzyme? We found in the present study that 5-LO can influence the formation and outgrowth of coherent tumor tissues as shown for the formation of MCTS from U-2 OS and HT-29 cells. These data are consistent with the large number of differentially expressed genes known to be involved in cell–cell contact and ECM formation we found in the 5-LO KO cells. Interestingly, pharmacological inhibition did not influence MCTS formation. Apparently, this is controlled by noncanonical functions of 5-LO.

The switch from epithelial to mesenchymal traits and back plays a fundamental role in tumor progression. During tumor initiation, a rigid epithelial cell morphology is important for the formation of a solid, encapsulated tumor body. In later stages, motile mesenchymal-like tumor cells invade the surrounding tissue and travel to distant locations where they change back to an epithelial type to establish a metastasis [87]. A large number of DEGs found in our RNAseq data are known to be involved in EMT, cell adhesion and cytoskeleton rearrangement. We therefore investigated the motility and invasive capacity of the 5-LO KO cells. From these data it became evident that directed motility towards a serum gradient considerably increased in absence of 5-LO in HCT-116 and U-2 OS KO cells. Pharmacological inhibition of 5-LO also triggered HCT-116 migration whereas U-2 OS migration was not influenced by the inhibitors. In contrast, haptotaxis assessed in a wound closure assay was not affected by 5-LO. Taken together, our data on cancer cell motility, spheroid formation and outgrowth suggest that expression of 5-LO fine tunes tumor cell motility and adherence thereby shaping the tumor establishment and metastasis. Again, this effect is highly cell type dependent.

Taken together, we found that the functional experiments using 5-LO inhibitors gave mixed results in the cell lines. This points to canonical as well as noncanonical properties of the enzyme which influence gene expression and cancer cell functions. Accordingly, 5-LO inhibitor treatment of the wild-type cell lines did only influence part of the DEGs found in the KO cells. 5-LO is known to physically interact with p53 and β-catenin thereby influencing their intracellular location and DNA binding [3, 86]. This might directly influence cell survival and proliferation. Furthermore, the enzyme was recently found to influence microRNA processing [88].

Considering the decidedly low levels of LTB_4_ and 5-HETE formed in the tumor cell lines, it is surprising that 5-LO products play a role in tumor cell functions at all. 5-LO does not accept phospholipid bound fatty acids but 5-HETE and 5-oxo-ETE can be found in cell membranes of activated human neutrophils. This suggests that 5-LO products can be directly re-incorporated into cell membranes after formation [89]. These oxygenated phospholipids are known to influence lipid bilayer fluidity and with it the activation of proteins in or attached to these membranes thereby controlling cell signaling, motility and death [90]. It is intriguing to speculate that cancer cells form low amounts of 5-LO products that control intracellular functions by the regulation of membrane fluidity instead of releasing these lipid mediators. It is also conceivable that low levels of intracellularly released 5-LO products are sufficient for the activation of nuclear receptors [91–93]. The mechanisms by which 5-LO directly influences the expression of certain genes is beyond the scope of the present manuscript and therefore has to be investigated in detail in future studies.

In addition to influence on functions and proliferation of tumor cells, 5-LO also regulated genes involved in inflammation and the manipulation of the tumor microenvironment among them MCP-1, fractalkine, PDGF, TGFβ_2_ and integrins [94–99]. This suggests that 5-LO is also involved in the recruitment and manipulation of the tumor stroma. It has been recently shown that 5-LO expression is upregulated in hypoxic areas of ovarian tumors. In this study 5-LO expression correlated with tumor invasiveness and tumor-associated macrophage numbers [100]. The important aspect of the interaction between 5-LO expressing tumors and associated stromal cells was beyond the scope of the present manuscript but will be addressed in follow-up studies by our group.

Based on our present findings, we conclude that presence of 5-LO affects the gene expression profile of cancer cells thereby influencing cell function and survival. We found that 5-LO participates in the coordination of tumor cell motility and proliferation as well as tumor cell invasion and MCTS formation by influencing the cytoskeleton as well as ECM components. This suggests that 5-LO plays a role during EMT and is involved in the maintenance of a solid tumor mass. Furthermore, the enzyme influences the expression of cytokines and cell adhesion factors important for the recruitment and manipulation of stromal cells such as tumor-associated macrophages and fibroblasts. This influence on gene expression and cell function seems to be in part exerted by 5-LO products although we found that 5-LO activity is rather low in HCT-116, HT-29 and U-2 OS cells. In addition to 5-LO-derived lipid mediators, noncanonical functions of the enzyme seem to play an important role.

Future studies should now focus on the following questions: (1) Which factors control expression and activity of 5-LO in cancer cells? (2) What is the mechanism behind 5-LO’s influence on gene regulation (noncanonical vs. canonical functions)? (3) Does tumor cell-derived 5-LO influence cells of the tumor stroma and what are the consequences?

## Supplementary information

Supplementary material

Supplementary Table 1

## Data Availability

The datasets generated and analyzed during the current study are available in the NCBI Sequence Read Archive (accession numbers GSE197947) repository.
