# Supplementary material for: Knock-out of 5-lipoxygenase in overexpressing tumor cells—consequences on gene expression and cellular function
Source: Cancer Gene Ther. 2022 Sep 16;30(1):108–23. doi: 10.1038/s41417-022-00531-9 (PMC9842508; doi:10.1038/s41417-022-00531-9)
Supplement: Supplementary file 1 — Supplementary material [file 41417_2022_531_MOESM1_ESM.docx]

## **Supplementary Information**

**Knock-out of 5-lipoxygenase in overexpressing tumor cells – consequences on gene expression and cellular function**

Hannah Weisser^a^, Tamara Göbel^a^, G Melissa Krishnathas^a^, Marius Kreiß^a^, Carlo Angioni^c^, Duran Sürün^b^, Dominique Thomas^c,d^, Tobias Schmid^e^, Ann-Kathrin Häfner^a^, Astrid S Kahnt^a^

^a^ Institute of Pharmaceutical Chemistry, Goethe University, Max-von-Laue-Straße 9, 60438 Frankfurt/Main, Germany.

^b^ Medical Systems Biology, Medical Faculty and University Hospital Carl Gustav Carus, TU Dresden, 01307 Dresden, Germany

^c^ Institute of Clinical Pharmacology, Pharmazentrum Frankfurt, ZAFES, Goethe University, Theodor-Stern-Kai 7, 60590 Frankfurt/Main, Germany.

^d^ Fraunhofer Institute of Translational Medicine and Pharmacology ITMP, Theodor-Stern-Kai 7, 60596 Frankfurt/Main, Germany.

^e^ Institute of Biochemistry I, Faculty of Medicine, Goethe University, Theodor-Stern-Kai 7, 60590 Frankfurt, Germany.

**SUPPLEMENTARY MATERIALS AND METHODS**

**Wound closure assay**

4.9 × 10^4^ cells were seeded in full growth medium in both chambers of an ibidi culture-insert 2 well system for 24-well plates (ibidi GmbH, Gräfelfing, Germany) and incubated at 37°C, 5% CO_2_ in a humidified atmosphere. After 24 hours, inserts as well as medium and non-adherent cells were removed and the cell layer was covered with medium again. Pictures were taken in the following days using a Zeiss Axio Vert.A1 microscope (Carl Zeiss Microscopy Deutschland GmbH, Oberkochen, Germany). The size of the gap was measured at 3 different locations for each time point employing the Zen blue software (version 2.6, Carl Zeiss Microscopy Deutschland GmbH, Oberkochen, Germany).

**WST-1 assay**

The cells were seeded in 96- well plates (3×104 cells/well). After 24 h, actinomycin D (3.5, 7, 10 and 14 nM) or etoposide (10, 20, 40, 50 µM) were added for 48 h. Control incubations received DMSO instead. Cell viability was assessed using the Cell Proliferation Reagent WST-1 (Roche Diagnostics) according to the manufacturer’s protocol and measured using an InfiniteR M200 plate reader (Tecan, Männedorf, Switzerland).

**Cell cycle analysis**

For cell cycle analysis, cells were seeded in serum starvation medium containing 0.5% FCS and incubated for 24 h (37°C, 5% CO_2_ in a humidified atmosphere). Then, medium was replaced by full growth medium. After 6 h, the cells were detached, separated using a cell strainer and fixed with 80% ethanol. All samples were then treated with 10 µg RNAseA and 10 µg propidium iodide for 30 minutes and measured by flow cytometry (BD FACSVerse™, BD Biosciences). Data were analyzed using the FlowJo software (version 10, BD Biosciences).

**SUPPLEMENTARY FIGURES AND TABLES**

**
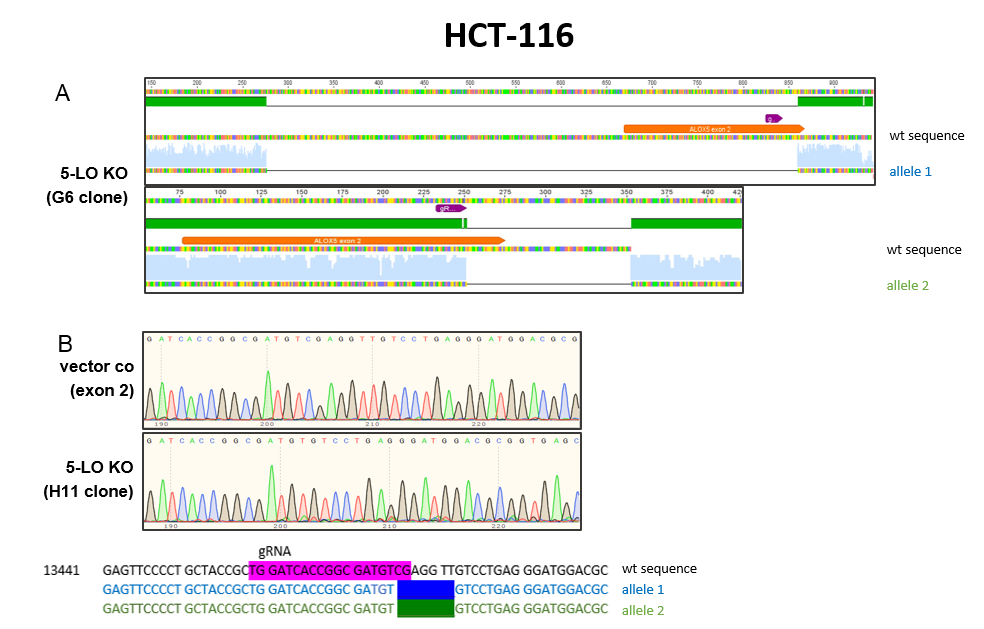
**

**Supplementary Figure 1**

**Validation of the 5-LO knockout in HCT-116 single cell clones** (A) Comparison of the sequencing data from control vector transfected HCT-116 cells with the HCT-116 KO clone G6 showing sequence alterations on both alleles. The gRNA binding site is marked in pink. Allele 1: Deletion of 583 bases (top); Allele 2: Substitution of 100 bases by 3 different bases (bottom). (B) Top: Comparison of the sequencing data from control vector transfected HCT-116 cells with the HCT-116 KO clone H11 showing sequence alterations on both alleles. Bottom: Comparison of the DNA wild type sequence with both alleles of the HCT-116 KO clone H11 carrying frameshift mutations. The gRNA binding site is marked in pink. Allele 1: Deletion of 7 bases (dark blue); Allele 2: Deletion of 7 bases (green).

**
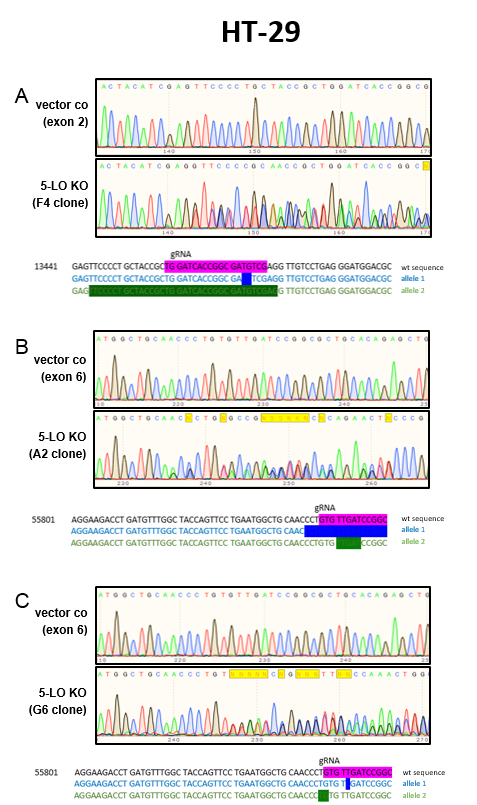
**

**Supplementary Figure 2**

**Validation of the 5-LO knockout in HT-29 single cell clones** (A) Top: Comparison of the sequencing data from control vector transfected HT-29 cells with the HT-29 KO clone F4 showing sequence alterations on both alleles. Bottom: Comparison of the DNA wild type sequence with both alleles of the HT-29 KO clone F4 carrying mutations. The gRNA binding site is marked in pink. Allele 1: Deletion of 2 bases (dark blue); Allele 2: Deletion of 36 bases (green). (B) Top: Comparison of the sequencing data from control vector transfected HT-29 cells with the HT-29 KO clone A2 showing sequence alterations on both alleles. Bottom: Comparison of the DNA wild type sequence with both alleles of the HT-29 KO clone A2 carrying frameshift mutations. The gRNA binding site is marked in pink. Allele 1: Deletion of 16 bases (dark blue); Allele 2: Deletion of 5 bases (green). (C) Top: Comparison of the sequencing data from control vector transfected HT-29 cells with the HT-29 KO clone G6 showing sequence alterations on both alleles. Bottom: Comparison of the DNA wild type sequence with both alleles of the HT-29 KO clone G6 carrying frameshift mutations. The gRNA binding site is marked in pink. Allele 1: Deletion of 1 base (dark blue); Allele 2: Deletion of 2 bases (green).

**
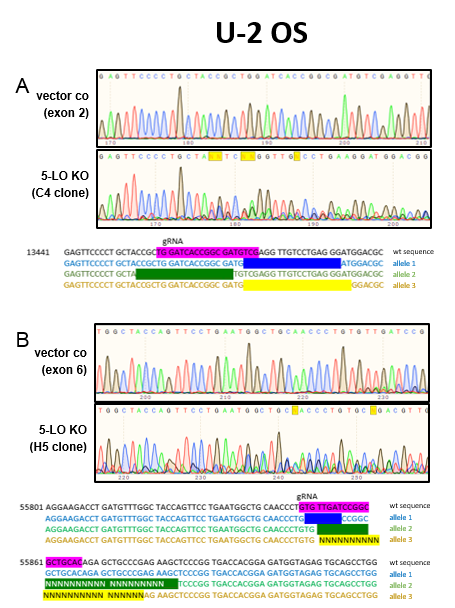
**

**Supplementary Figure 3**

**Validation of the 5-LO knockout in U-2OS single cell clones** (A) Top: Comparison of the sequencing data from control vector transfected U-2OS cells with the U-2OS KO clone C4 showing sequence alterations on all three alleles. Bottom: Comparison of the DNA wild type sequence with all three alleles of the U-2OS KO clone C4 carrying mutations. The gRNA binding site is marked in pink. Allele 1: Deletion of 18 bases (dark blue); Allele 2: Deletion of 18 bases (green); Allele 3: Deletion of 20 bases (yellow). (B) Top: Comparison of the sequencing data from control vector transfected U-2OS cells with the U-2OS KO clone H5 showing sequence alterations on all three alleles. Bottom: Comparison of the DNA wild type sequence with all three alleles of the U-2OS KO clone H5 carrying frameshift mutations. The gRNA binding site is marked in pink. Allele 1: Deletion of 7 bases (dark blue); Allele 2: Substitution of 34 bases by 20 different ones (green); Allele 3: Substitution of 28 bases by 28 different ones (yellow).

**
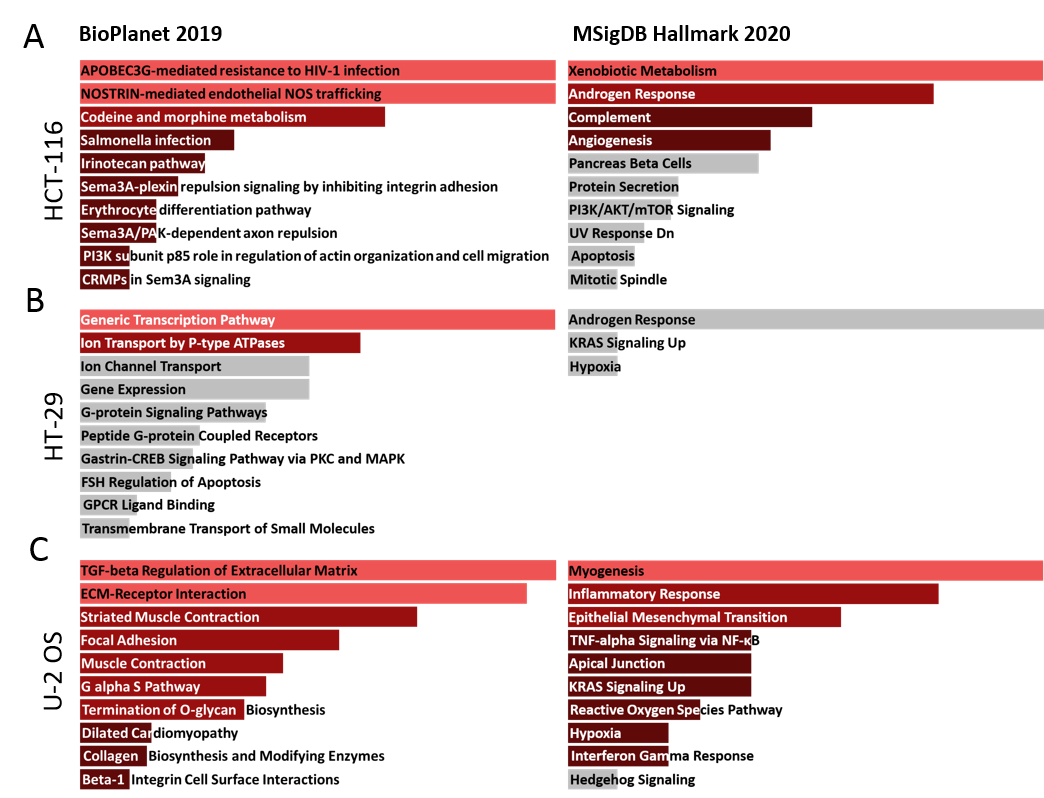
**

**Supplementary Figure 4**

**Gene set enrichment analyses of the differentially regulated genes after 5-LO KO.** Each KO clone and the corresponding empty vector control were measured in three biological replicates during genome-wide RNA sequencing. Genes showing a log2-fold change > 1 and an adjusted p-value < 0.05 compared to the empty vector control in each KO clone of a respective cell line were considered differentially expressed. (A-C) Gene set enrichment analysis of the sequencing data was performed employing the NCATS BioPlanet 2019 and GSEAs MSigDB Hallmark 2029 tools.


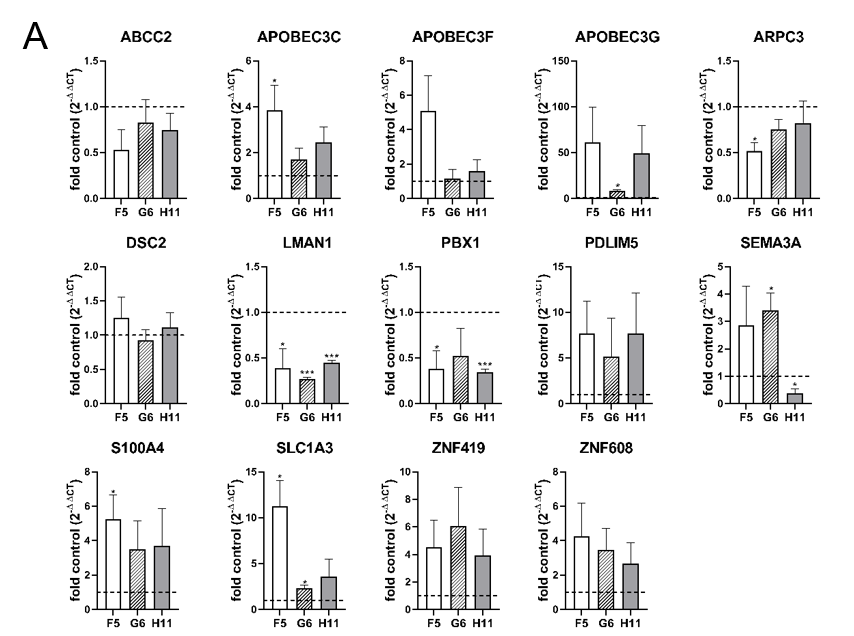


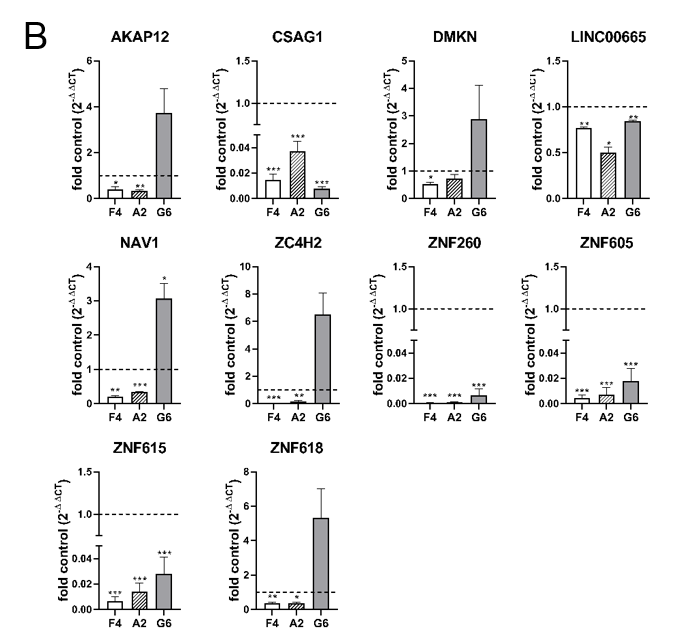


**Supplementary Figure 5**

**RT-qPCR analysis of selected genes in HCT-116 and HT-29 cells after 5-LO KO.** mRNA expression of a selection of differentially regulated genes is presented. (A) HCT-116, (B) HT-29 cells. Gene expression was normalized to *ACTB* (house keeping gene) and the corresponding control vector cells (2^-ΔΔct^ method). Data are presented as mean + SD of 3 independent experiments. Asterisks indicate significant changes vs. control vector cells. *P ≤ 0.05, **P ≤ 0.01, ***P ≤ 0.001.

**
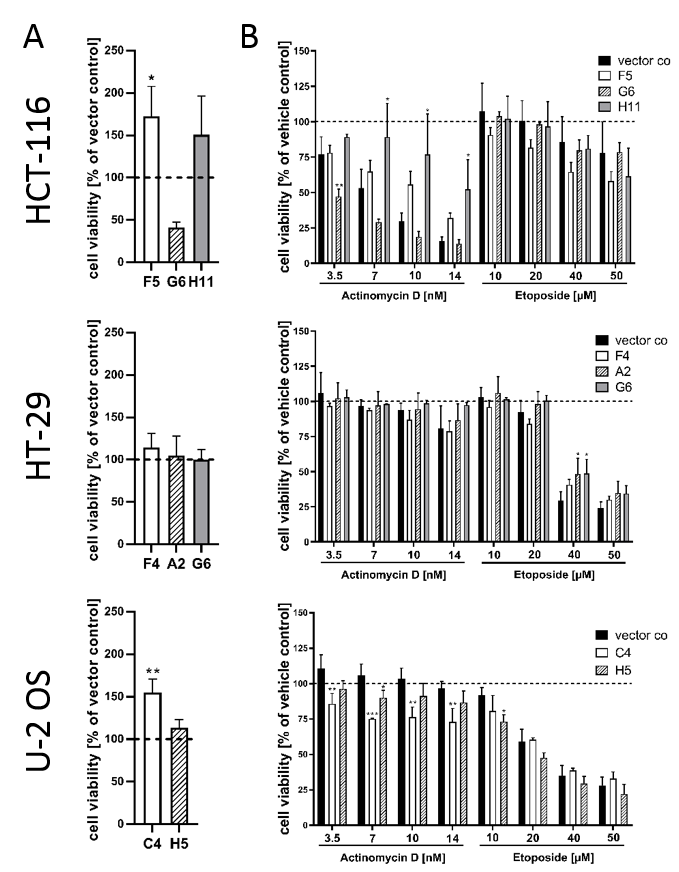
**

**Supplementary Figure 6**

**Influence of the 5-LO knockout on cell viability.** (A) Cell viability of the 5-LO KO clones was assessed after 72 h using the WST-1 assay. Viability is depicted as % of the vector control. (B) Cell viability of 5-LO KO clones and vector control cells after treatment with the cytotoxic drugs actinomycin D (3.5, 7, 10, 14 nM) and etoposide (10, 20, 40, 50 µM) for 48 h. Cell viability was assessed using the WST-1 assay. Data are depicted as % DMSO treated control.


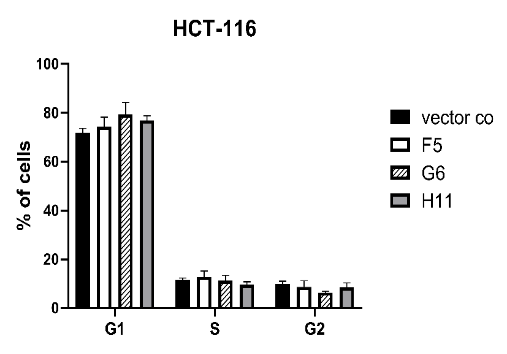

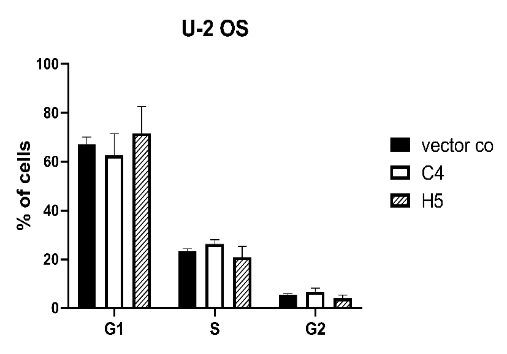

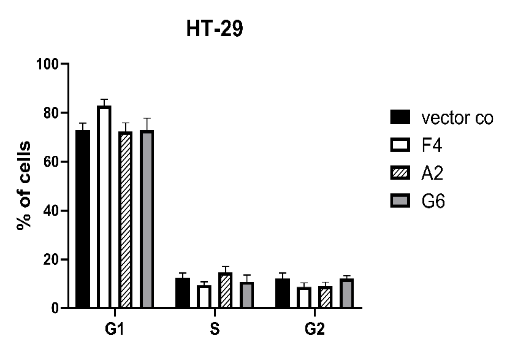


**Supplementary Figure 7**

**Cell cycle analysis of the 5-LO KO cells.** Cells were synchronized by serum starvation in medium containing 0.5% FCS for 24 h. Then, the medium was changed to full growth medium containing 10% FCS. After 6 h, the cells were harvested, fixed and analyzed via Flow cytometry. Data are presented as mean + SD of 3 independent experiments.


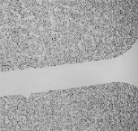

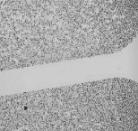

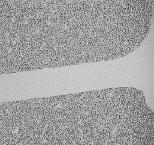

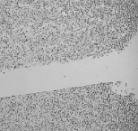

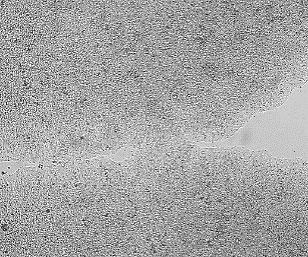

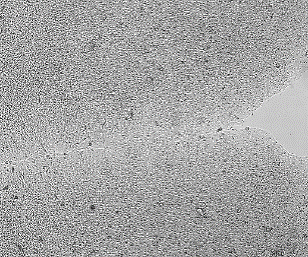

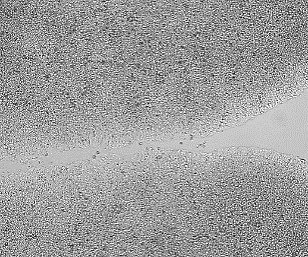

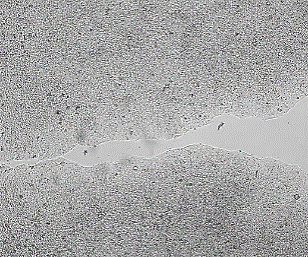


**HT-29**

vector

co

F4

A2

G6

0 h


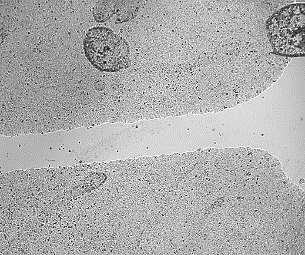

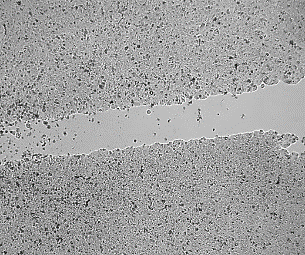

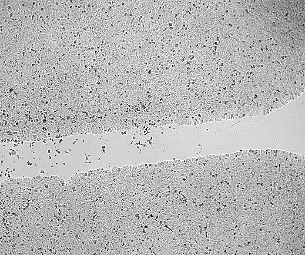

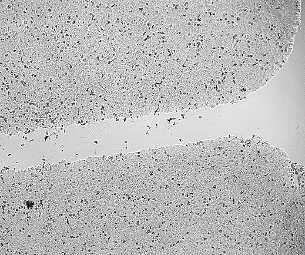


48 h

120 h


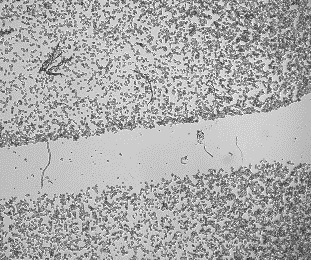

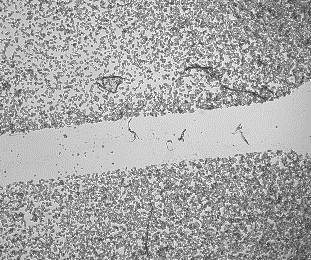

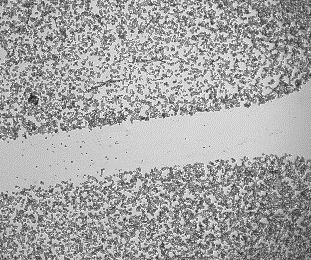

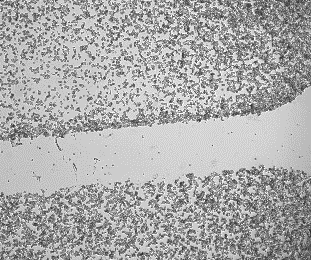

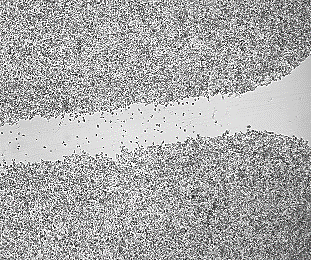

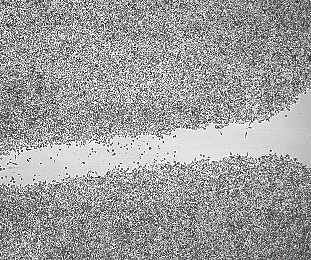

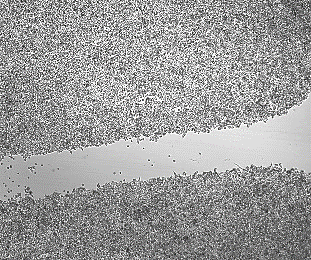

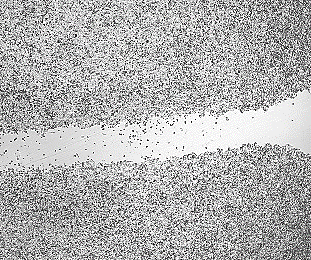

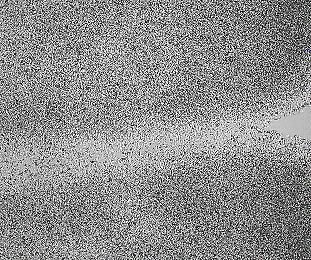

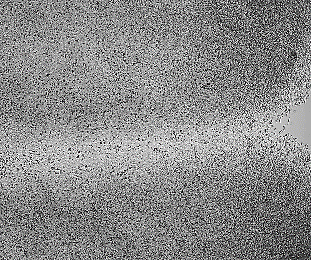

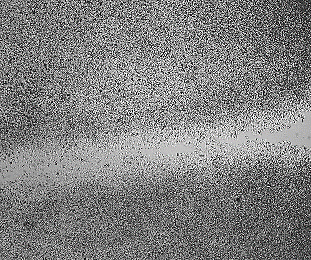

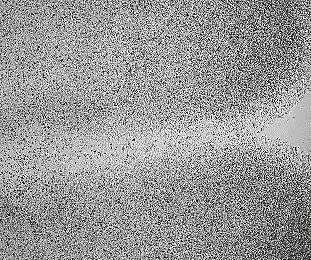


vector

co

F5

G6

H11

**HCT-116**

0 h

32 h

66 h


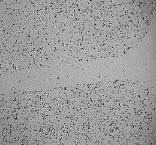

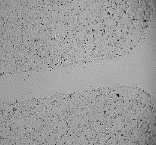

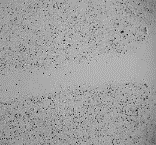

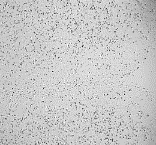

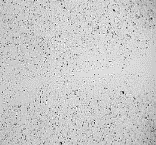

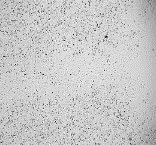

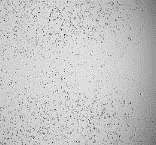

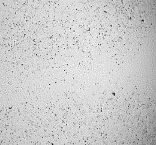

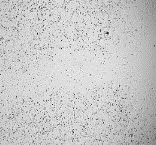


vector

co

C4

H5

**U-2 OS**

0 h

13 h

16 h

**Supplementary Figure 8**

**Wound closure in 5-LO KO cells.** Closing of an artificial wound gap yielded by seeding cells in 2-well culture-inserts. Cells were allowed to attach for 24 h, inserts were removed, the cell layer was washed carefully with PBS to remove all unattached cells and finally covered with fresh medium. Pictures were taken in the following using a Zeiss Axio Vert.A1 microscope (Carl Zeiss Microscopy Deutschland GmbH, Oberkochen, Germany).

| Gene | clone | | | | Gene | clone | | | | Gene | clone | | | |
| --- | --- | --- | --- | --- | --- | --- | --- | --- | --- | --- | --- | --- | --- | --- |
|  | **C4** | | **H5** | |  | **C4** | | **H5** | |  | **C4** | | **H5** | |
| **× control**  **[2^-ΔΔCт^]** | **mean** | **SD** | **mean** | **SD** | **× control**  **[2^-ΔΔCт^]** | **mean** | **SD** | **mean** | **SD** | **× control**  **[2^-ΔΔCт^]** | **mean** | **SD** | **mean** | **SD** |
| **ACKR3** | 6.92 | 2.58 | 2.79 | 1.13 | **FMNL2** | 4.73 | 6.60 | 0.53 | 0.61 | **PDE4B** | 0.17 | 0.04 | 0.15 | 0.08 |
| **ADGRG1** | 0.32 | 0.09 | 0.48 | 0.04 | **GALM** | 3.87 | 1.37 | 3.53 | 1.30 | **PDGFA** | 0.58 | 0.28 | 0.35 | 0.08 |
| **ADGRL3** | 0.25 | 0.07 | 0.28 | 0.04 | **GPR132** | 0.01 | 0.008 | 0.008 | 0.009 | **PDLIM1** | 1.52 | 0.52 | 1.84 | 0.76 |
| **ADGRL4** | 0.36 | 0.16 | 0.19 | 0.08 | **HAS2** | 0.22 | 0.05 | 0.41 | 0.14 | **PLAC1** | 4.61 | 1.66 | 5.27 | 1.19 |
| **ADIRF** | 0.76 | 0.20 | 0.87 | 0.11 | **HES7** | 0.57 | 0.16 | 0.88 | 0.56 | **PLAC8** | 3.24 | 0.46 | 4.46 | 0.56 |
| **ALDH1L1** | 0.40 | 0.12 | 0.15 | 0.04 | **HIST1H2AC** | 0.79 | 0.25 | 0.86 | 0.13 | **PRR16** | 0.29 | 0.09 | 0.35 | 0.08 |
| **ANK3** | 0.63 | 0.19 | 0.16 | 0.04 | **HSPA1A** | 1.07 | 0.15 | 1.22 | 0.36 | **PRSS33** | 0.38 | 0.41 | 0.40 | 0.47 |
| **ARHGEF4** | 0.20 | 0.08 | 0.24 | 0.04 | **HSPB2** | 6.75 | 2.95 | 18.49 | 6.09 | **RGS16** | 0.19 | 0.10 | 0.05 | 0.02 |
| **ASXL3** | 1.01 | 0.15 | 0.70 | 0.23 | **IFI44** | 0.19 | 0.02 | 0.02 | 0.004 | **RGS4** | 0.39 | 0.06 | 0.31 | 0.04 |
| **BCL2A1** | 0.13 | 0.06 | 0.10 | 0.07 | **IFI44L** | 0.24 | 0.06 | 0.10 | 0.006 | **RIPOR2** | 7.38 | 4.59 | 8.09 | 3.78 |
| **BHLHE41** | 0.24 | 0.06 | 0.43 | 0.10 | **IGFBP7** | 0.29 | 0.06 | 0.76 | 0.16 | **SELENBP1** | 0.04 | 0.03 | 0.01 | 0.005 |
| **BMPR1B** | 0.36 | 0.07 | 0.60 | 0.20 | **IGFL1** | 16.98 | 13.96 | 29.29 | 18.89 | **SERPINF1** | 0.09 | 0.02 | 0.01 | 0.004 |
| **BST2** | 0.11 | 0.03 | 0.01 | 0.002 | **IGFL3** | 0.16 | 0.15 | 0.26 | 0.22 | **SLC6A17** | 0.26 | 0.09 | 0.19 | 0.01 |
| **C1orf198** | 2.10 | 0.10 | 3.65 | 0.52 | **KCNB1** | 2.01 | 0.49 | 3.36 | 2.06 | **SLC25A25** | 0.98 | 0.42 | 1.20 | 0.11 |
| **CDH6** | 0.65 | 0.28 | 0.19 | 0.05 | **LAMC2** | 0.33 | 0.09 | 0.52 | 0.06 | **SLFN5** | 0.46 | 0.20 | 0.43 | 0.15 |
| **CFI** | 0.17 | 0.08 | 0.03 | 0.01 | **LEMD1** | 0.18 | 0.06 | 0.19 | 0.03 | **SLITRK6** | 0.07 | 0.03 | 0.03 | 0.07 |
| **CHN1** | 0.36 | 0.05 | 0.32 | 0.04 | **LIMS2** | 2.45 | 0.39 | 2.45 | 0.48 | **SNX13** | 0.49 | 0.05 | 0.53 | 0.06 |
| **CNN1** | 3.09 | 0.93 | 9.50 | 3.50 | **LIPH** | 0.25 | 0.11 | 0.14 | 0.05 | **SPANXB1** | 5.09 | 1.06 | 11.70 | 4.23 |
| **COL8A1** | 0.22 | 0.04 | 0.44 | 0.08 | **LLGL2** | 21.83 | 15.37 | 21.78 | 16.14 | **SPP1** | 0.22 | 0.04 | 0.12 | 0.06 |
| **COL20A1** | 0.66 | 0.21 | 0.45 | 0.15 | **LSP1** | 2.18 | 0.41 | 2.19 | 0.05 | **SRGN** | 0.08 | 0.01 | 0.10 | 0.03 |
| **CPA4** | 2.11 | 0.23 | 3.40 | 0.82 | **LY6KI** | 2.29 | 0.38 | 5.11 | 0.35 | **STARD5** | 0.42 | 0.01 | 0.59 | 0.10 |
| **CRAT** | 5.52 | 0.99 | 6.68 | 1.39 | **MAP2K6** | 0.33 | 0.06 | 0.16 | 0.06 | **SULT1A1** | 1.10 | 0.06 | 1.24 | 0.20 |
| **DDIT4** | 0.43 | 0.29 | 0.40 | 0.21 | **MGST1** | 0.27 | 0.01 | 0.35 | 0.06 | **SYT8** | 1.9 | 0.45 | 7.18 | 3.64 |
| **DOCK4** | 0.52 | 0.21 | 0.68 | 0.15 | **MISP** | 3.10 | 0.66 | 2.18 | 0.34 | **TMEM40** | 2.72 | 0.26 | 3.31 | 0.13 |
| **DRAXIN** | 0.25 | 0.07 | 0.14 | 0.06 | **MLLT3** | 0.59 | 0.06 | 0.54 | 0.19 | **TMEM154** | 0.29 | 0.18 | 0.12 | 0.05 |
| **EDN2** | 8.86 | 6.46 | 16.49 | 10.42 | **MOB3B** | 1.33 | 0.27 | 2.95 | 0.62 | **TMEM200A** | 0.27 | 0.12 | 0.29 | 0.10 |
| **ETV1** | 0.20 | 0.08 | 0.001 | 0.001 | **MYL2** | 3.57 | 1.12 | 5.56 | 1.00 | **TNFAIP6** | 0.06 | 0.03 | 0.04 | 0.02 |
| **EVI2A** | 0.27 | 0.18 | 0.45 | 0.39 | **NOV** | 0.39 | 0.04 | 0.29 | 0.06 | **TOX** | 0.54 | 0.14 | 0.32 | 0.07 |
| **FAM78A** | 3.72 | 3.77 | 2.5 | 2.57 | **OSCAR** | 0.52 | 0.36 | 0.34 | 0.28 | **TRBC2** | 0.07 | 0.02 | 0.04 | 0.01 |
| **FAP** | 0.31 | 0.09 | 0.33 | 0.06 | **PAM** | 0.61 | 0.18 | 0.60 | 0.01 | **VGF** | 0.55 | 0.31 | 0.46 | 0.02 |
| **FGD4** | 0.89 | 0.11 | 0.76 | 0.16 | **PAX7** | 1.49 | 0.46 | 2.97 | 0.41 | **XYLT1** | 0.42 | 0.08 | 0.62 | 0.13 |

**Suppl. table 2** **RT-qPCR analysis of selected genes in U-2 OS cells after 5-LO KO.** mRNA expression of a selection of differentially regulated genes is presented. Gene expression was normalized to ACTB (housekeeping gene) and the corresponding control vector cells (2^-ΔΔct^ method). Data are presented as mean + SEM of 3 independent experiments.
